# A comprehensive review on the carcinogenic potential of bisphenol A: clues and evidence

**DOI:** 10.1007/s11356-021-13071-w

**Published:** 2021-03-05

**Authors:** Nadeem Ghani Khan, Jacinta Correia, Divya Adiga, Padmalatha Satwadi Rai, Herman Sunil Dsouza, Sanjiban Chakrabarty, Shama Prasada Kabekkodu

**Affiliations:** 1 Department of Cell and Molecular Biology, Manipal School of Life Sciences, Manipal Academy of Higher Education, Manipal, Karnataka 576104 India; 2 Department of Biotechnology, Manipal School of Life Sciences, Manipal Academy of Higher Education, Manipal, Karnataka 576104 India; 3 Department of Radiation Biology and Toxicology, Manipal School of Life Sciences, Manipal Academy of Higher Education, Manipal, Karnataka 576104 India; 4 Center for DNA repair and Genome Stability (CDRGS), Manipal Academy of Higher Education, Manipal, Karnataka 576104 India

**Keywords:** Bisphenol A,, Endocrine disruptor,, Carcinogen,, Environmental toxicant,, Human cancer

## Abstract

Bisphenol A [BPA; (CH_3_)_2_C(C_6_H_4_OH)_2_] is a synthetic chemical used as a precursor material for the manufacturing of plastics and resins. It gained attention due to its high chances of human exposure and predisposing individuals at extremely low doses to diseases, including cancer. It enters the human body via oral, inhaled, and dermal routes as leach-out products. BPA may be anticipated as a probable human carcinogen. Studies using in vitro cell lines, rodent models, and epidemiological analysis have convincingly shown the increasing susceptibility to cancer at doses below the oral reference dose set by the Environmental Protection Agency for BPA. Furthermore, BPA exerts its toxicological effects at the genetic and epigenetic levels, influencing various cell signaling pathways. The present review summarizes the available data on BPA and its potential impact on cancer and its clinical outcome.

## Introduction

Bisphenol A (BPA) is an endocrine-disrupting synthetic chemical used to manufacture consumer products such as water bottles, water pipes, and food cans. It is one of the most abundant industrial synthetic chemicals produced globally (more than 6  ×  109 lb/year) (Gao et al. 2015). BPA was synthetized by Alexander Dianin and has been commercially available since 1957 (Hoque 2019). BPA is a colorless, solid organic compound with the chemical formula (CH_3_)_2_C(C_6_H_4_OH)_2_. It is a 4,4’-methanediyldiphenol with moderate solubility in water, whereas it is completely soluble in an organic solvent. BPA is widely present in hard plastics, epoxy resins, medical devices, dental sealants, baby toys, kitchenware, thermal receipt paper, and internal coatings in food and beverage packing cans/containers (Kubwabo et al. 2009). Thus, BPA has ubiquitously been found in the domestic environment around the world.

Exposure to BPA is a major health concern due to its ability to disrupt endocrine signaling pathways and cause varieties of human diseases even at very low doses (den Braver-Sewradj et al. 2020). BPA is a derivative of diphenylmethane containing two hydroxyphenyl groups owing to its structural similarity to synthetic estrogen. Many studies have reported that BPA mimics and competes with estrogen to bind to the estrogen receptors α and β (ERα and ERβ) and modulate estrogen-responsive gene expression (Paris et al. 2002; Lee et al. 2012). Pupo et al. demonstrated that BPA activates G protein-coupled estrogen receptor 1 (GPER, ERK 1/2, EGFR) signaling pathway in cancer cells via inducing the expression of target genes coupled with G protein receptor (Pupo et al. 2012). *In vitro* and *in vivo* studies have shown that BPA exposure has a pro-carcinogenic influence in hormone-dependent and hormone-independent cancers (Gao et al. 2015; Seachrist et al. 2016). BPA exposure is reported to alter the cancer cells’ biological behaviors, notably, proliferation, invasion, growth, survival, migration, and apoptosis (Chevalier et al. 2012; Prins et al. 2014; Ge et al. 2014; Wang et al. 2015, 2017, 2019; Ma et al. 2015; Song et al. 2015; Pfeifer et al. 2015; Shi et al. 2017; Jeong et al. 2017; Sauer et al. 2017; Li et al. 2017; Huang et al. 2018; Hui et al. 2018; Qu et al. 2018; Hanafi et al. 2019). Besides, in vitro studies have shown that low doses of BPA exposure have reported inducing resistance to anticancer drugs such as tamoxifen (TAM), carboplatin, poly ADP ribose polymerase (PARP) inhibitors, doxorubicin, bevacizumab, vinblastine, cisplatin, and others by modulating the expression of many oncogenic signaling pathways in both hormone-dependent and hormone-independent human cancers (Hafezi and Abdel-Rahman 2019). MAPK, PI3K/AKT, NFκB and JNK, and Ca^2+^ homeostasis are the most widely studies pathways in relation to BPA and cancer (Zhang et al. 2016; Qu et al. 2018). The current experimental evidence demonstrates that BPA can significantly increase the risk of both hormone-dependent and hormone-independent cancers via inducing epigenetic changes such as altered promoter methylation of tumor suppressors or oncogenes, global hypomethylation, and histone modifications and by modulating the expression of noncoding RNAs such as miRNA, lncRNA, and snoRNA (Ho et al. 2015; Şenyildiz and Özden 2015; Şenyıldız et al. 2016; Senyildiz et al. 2017; Prins et al. 2017; Cheong et al. 2018; Fatma Karaman et al. 2019). Herein, we review the current literature on the role of BPA in hormone-dependent and hormone-independent human cancers, their mechanism of action, and their potential impact on cancer development.

## Human exposure, metabolism, and mechanism of action

**Fig. 1:**
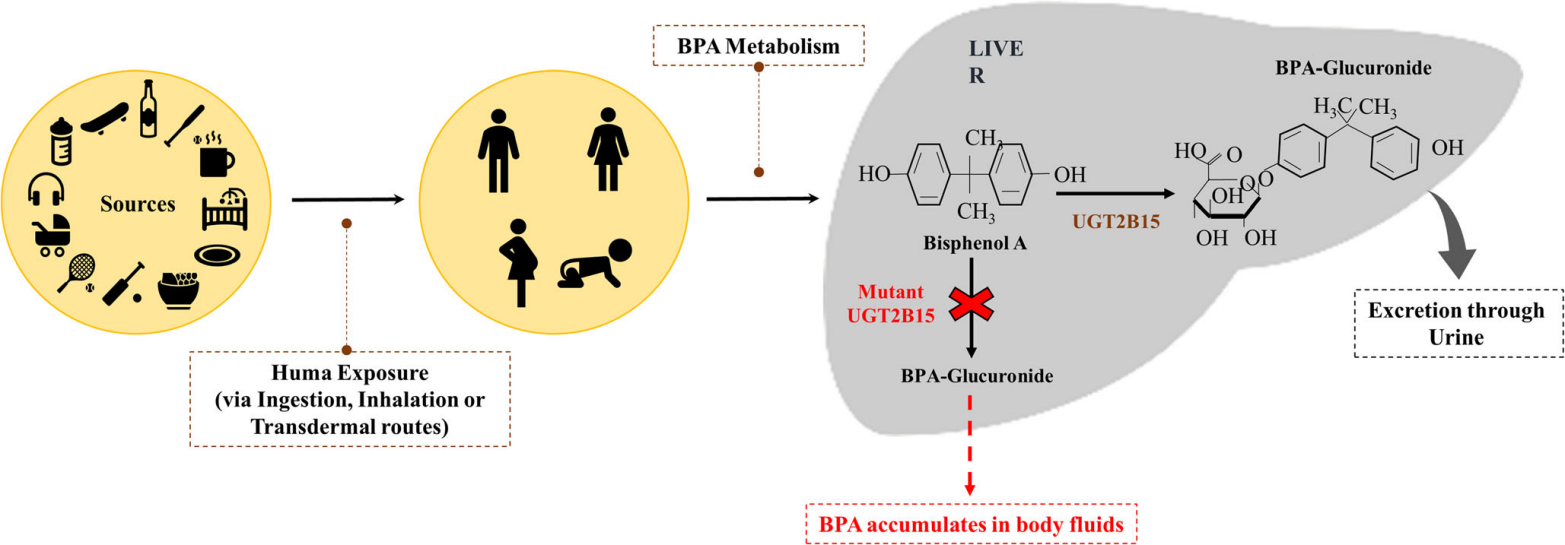
Bisphenol A exposure to humans and its metabolism**.** BPA is metabolized in the liver through glucuronidation. The liver enzyme UDP-glucuronosyltransferases 2B15 (UGTs) is responsible for the glucuronidation of BPA followed by its excretion through sweat or urine in the form of BPA glucuronide. Deregulated activity of this enzyme results in BPA accumulation leading to aberrant oncogenic signaling

Human exposure to BPA occurs when it leaches from plastic-lined food and drink containers, water bottles, and dental sealants when they are repeatedly heated or washed with harsh detergents, or when they contain acidic liquids. It enters the human body via inhalation, ingestion (consumption of contaminated food and water), and through dermal exposure (TSAI 2006). BPA is absorbed by the gastrointestinal tract and gets metabolized in the liver through glucuronidation or sulfonation. The liver enzyme UDP-glucuronosyltransferases 2B15 (UGTs) is responsible for the glucuronidation of BPA, followed by its excretion from the body either through bile or urine in the form of BPA glucuronide (Kurebayashi 2003; Genuis et al. 2012; Thongkorn et al. 2019). The abnormalities in UGTs enzyme lead to the elevation of unconjugated BPA concentration in the body. Many studies have reported the presence of the unconjugated forms of BPA in human body fluids, such as milk, maternal urine, amniotic, and placental fluids, and in neonates that corresponds with the occurrence of many hormonal abnormalities (Vandenberg et al. 2010; Inadera 2015) (Fig. 1).

**Fig. 2:**
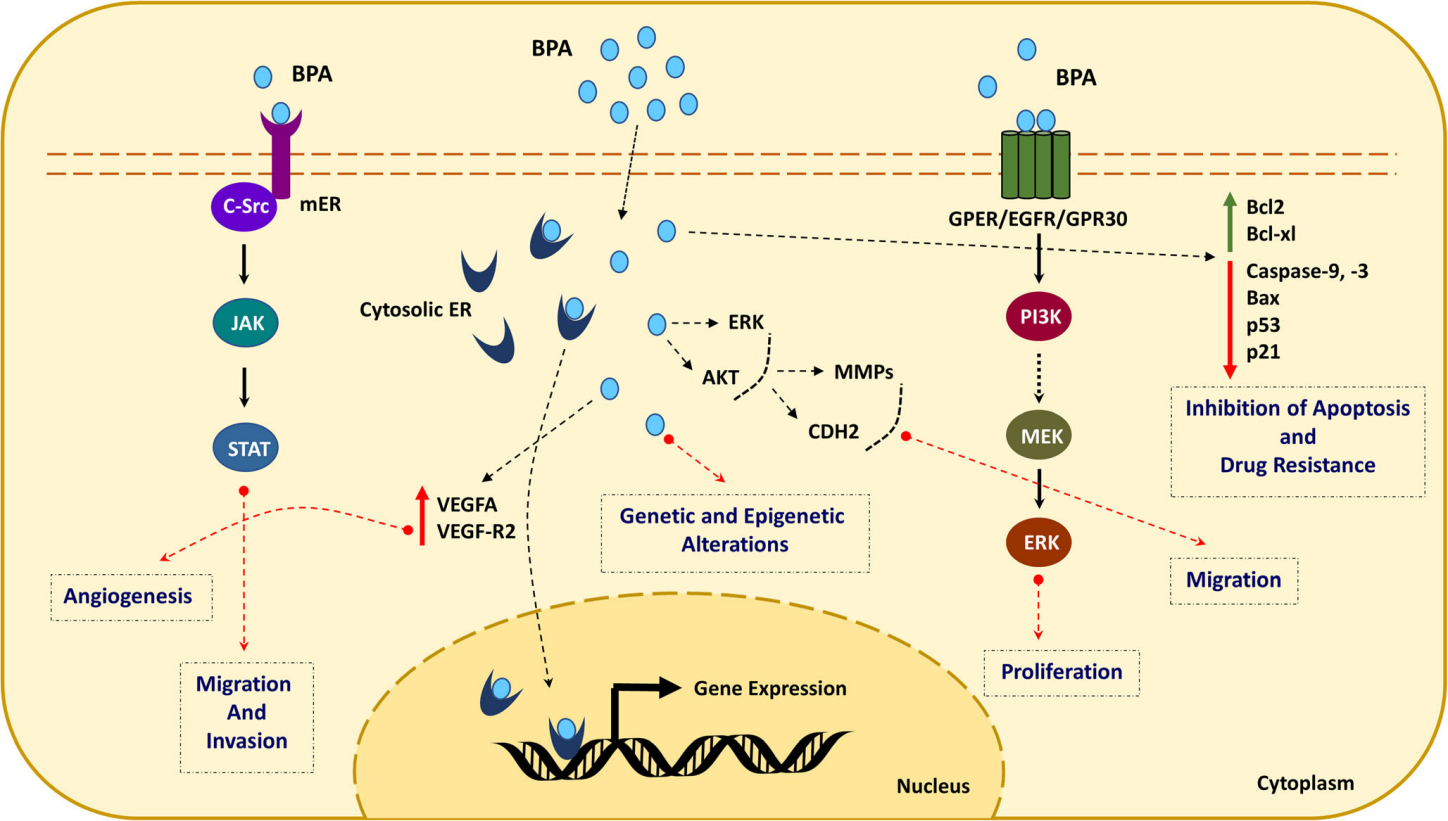
Mechanism of action of bisphenol A and associated cancer hallmarks. Mechanism of BPA-stimulated carcinogenesis may be due to its estrogenic activity. BPA binds to membrane estrogen receptors (mERs), nuclear ERs, and receptor GPR30 and alters the genomic and non-genomic signaling pathways differently in different cell types and alters the normal biological functions

BPA exposure affects several signaling pathways, including interference with cellular receptors (nuclear, steroid hormone, and orphan) functioning. Several studies have demonstrated estrogen receptors, androgen receptor, estrogen-related receptors, thyroid hormone receptor, peroxisome proliferator-activated receptors, pregnane X receptor, and aryl hydrocarbon receptor and downstream signaling as targets of BPA. Besides, many enzymatic pathways related to steroid biosynthesis and metabolism associated with endocrine and/or reproductive systems are targeted by BPA. Modulation of these pathways has been linked to cancer development. For example, abnormal expression of estrogen receptors plays an essential role in the development of carcinoma of the breast, ovary, liver, and low-grade endometrium (Gao et al. 2015). BPA has a higher affinity to bind to various cell surface receptors such as ER (ERα and ERβ), androgen receptor (AR), membrane receptor GPER (GPR30), epidermal growth factor receptor (EGFR), and estrogen-related receptors (ERRs) (Acconcia et al. 2015; Gao et al. 2015). The primary mechanism of BPA-stimulated carcinogenesis may be due to its estrogenic activity. BPA binds to membrane estrogen receptors (mERs), nuclear ERs, and receptor GPR30 and alters the genomic and non-genomic signaling pathways differently in different cell types and alters the normal biological functions leading to carcinogenesis (Wang et al. 2010). Taken together, BPA acts through both estrogen-dependent and estrogen-independent pathways in cancer (Fig. 2). The sections below describe the association between BPA and cancer.

## Association of BPA with cancer

**Fig. 3:**
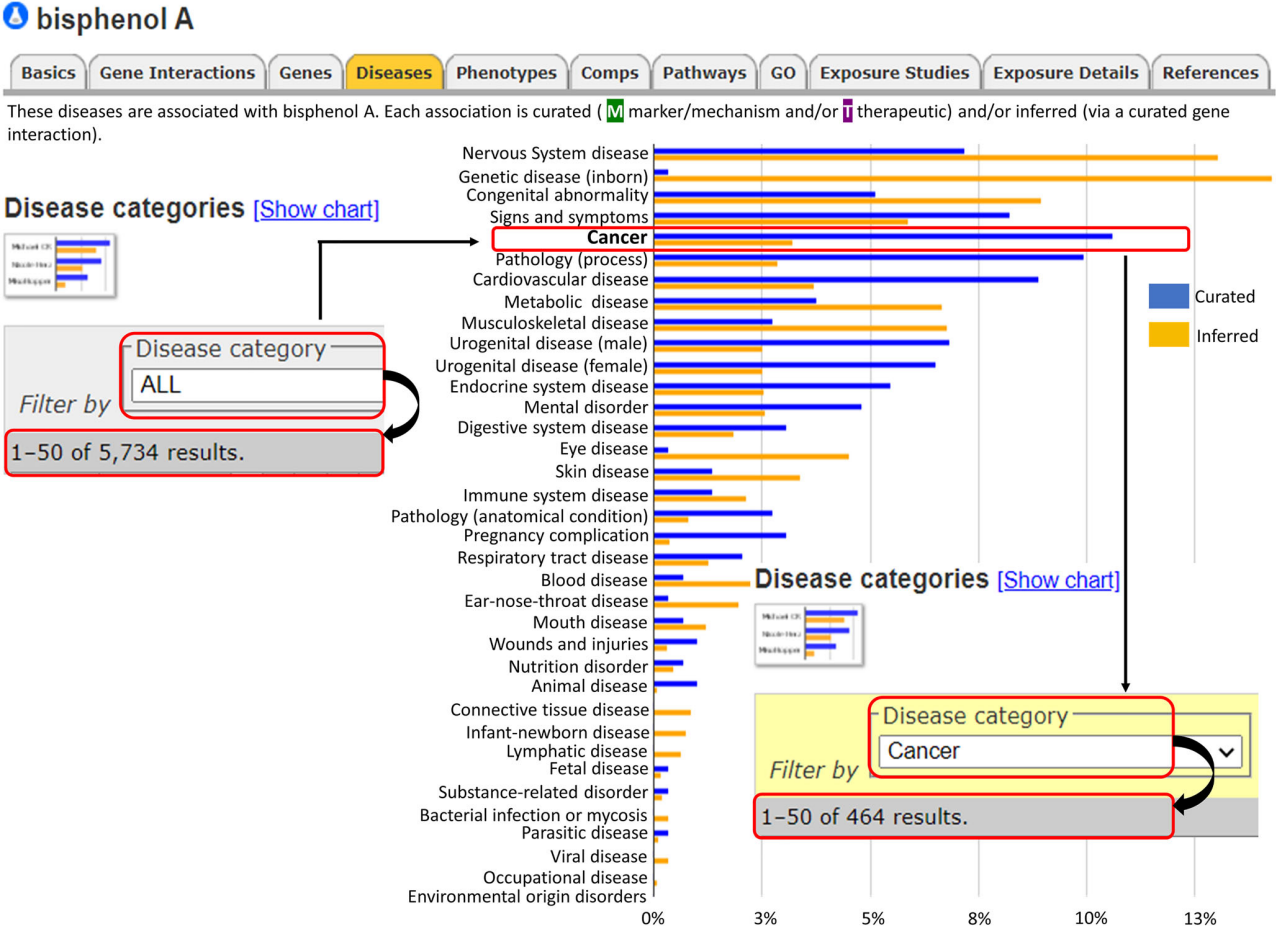
Bisphenol A and associated diseases (CTD disease landscape). Across 36 different groups, BPA is associated with 5735 diseases. Among the cancer classes, bisphenol A is linked with 466 of the cancers

**Fig. 4:**
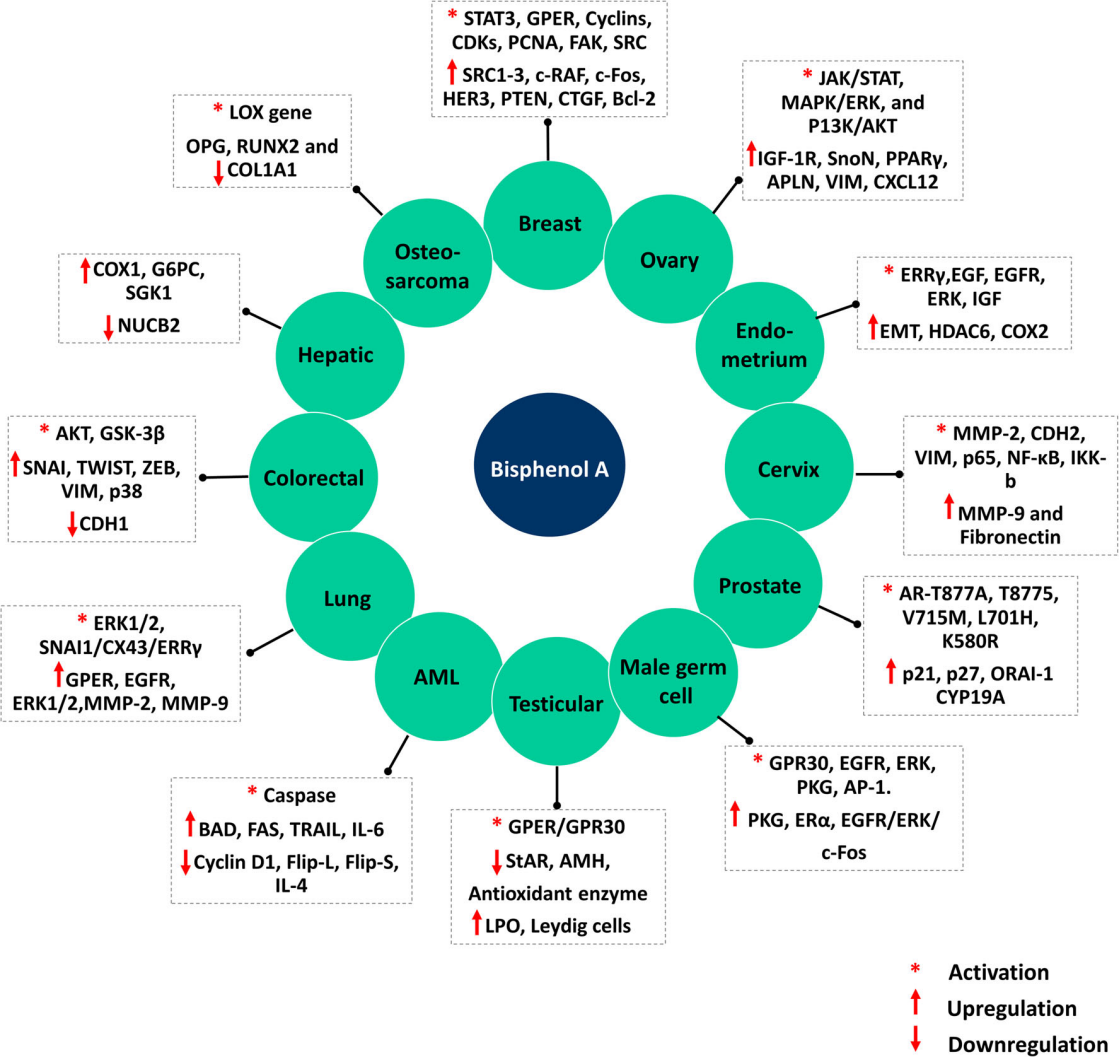
Role of bisphenol A in the development of hormone-related cancers. BPA to facilitate the acquisition of cancer hallmarks via modulating the expression of many oncogenic signaling pathways

We have used comparative toxicogenomics database (CTD; http://ctdbase.org/) to predict the association of BPA with human health (Davis et al. 2021). Analysis using CTD identified association of BPA with 5734 diseases across 36 disease categories. Interestingly, among cancer classes, BPA was associated with 464 entries (Fig. 3). Exposure to BPA has been linked with increased cancer risk (Gao et al. 2015). The carcinogenic potential of BPA was demonstrated in several previously published studies using in vitro and in vivo models. A study by Seachrist and co-workers in 2016 showed that early-life exposure to BPA is a risk factor for breast and prostate cancer and proposed to classify BPA as a chemical carcinogen belonging to “group 2A” (Seachrist et al. 2016). There are numerous evidences to demonstrate that exposure to BPA, even at a very low level, can either increase cancer risk or aggravate cancer. Some of the hormone-dependent cells are highly sensitive to BPA as it induces pro-proliferative pathways. Several earlier studies demonstrated the pro-carcinogenic activity of BPA. Both in vitro, in vivo, and animal studies have shown the ability of BPA to facilitate the acquisition of cancer hallmarks via altering the biological behavior of the cells and induction of pro-carcinogenic signaling pathways. As per the literature, BPA can induce proliferation, growth, migration, and invasion in various cell types such as cervical (SiHa, HeLa, C33A), breast (MCF7, MDA-MB-231, BT-549), prostate (LNCaP), human trophoblasts (HTR-8/SVneo), mesenchymal stem cells (hUM-MSCs), ovarian (OVCAR-3, SkBr3), lung (A549), and colorectal (SW480) via activation of signaling pathways, notably JAK-STAT, PI3-AKT, MAPK, and others (Wang et al. 2013; Nomiri et al. 2019). In addition to its carcinogenic role, several studies have demonstrated the genotoxic effect of BPA using in vitro systems. By modulating the epigenetic enzymes, BPA can bring about genome-wide epigenetic changes leading to altered expression. The epigenetic changes induced upon exposure to BPA are suspected of playing a key role in disease pathophysiology, including cancer. The following section describes the association of BPA with different cancer types (Table 1 and Fig. 4).

**Table 1:** Mechanism of action of bisphenol A on various cancers

Cancer	Targets	Hallmarks	References
Breast cancer	• Activation of STAT3, GPER, Cyclins (A, D3), CDKs (2 and 6), PCNA, FAK, SRC, ERK1/2 • Upregulation of GPER, EGFR, PR-A, SRC1-3, AKT, c-RAF, ERK1/2, AKT, c-Fos, HER3, PTEN, ERRγ, P38, MMP-2, MMP-9, CTGF, Bcl-2 • Downregulation of FOXA1, Fork head Family Transcription Factor, P53, BAX and BIM, PDCD5 and BCL2L11 • Hypermethylation of BCL2L11, PARD6G, FOXP1, and SFRS11, NUP98, and CtIP (RBBP8) • Decreases the expression of TET2 among the three TET dioxygenases. Decrease the level of genomic 5hmC	• Increases proliferation, migration, and invasion in vitro and induces epithelial-mesenchymal transition (EMT) • Increase in the levels of progesterone receptors • Reduction in the efficacy of multiple chemotherapeutic agents • Emphasizing the pathway of ER receptor–DNMTs-TET2-DNA hydroxymethylation	(Zhang et al. 2014, LaPensee et al. 2009, Pupo et al. 2012, Dairkee et al. 2013, Wang et al. 2015)
Ovarian cancer	• Activates JAK/STAT, MAPK/ERK, and PI3K/AKT • Phosphorylates IRS, CCND1, • Upregulates mRNA levels of ERα, IGF-1R, SnoN, PPARγ, APLN, VIM, CXCL12 • Downregulates SMAD3, CDH1, ZO-1 • Inhibits TGF-β, CASP3, CASP7, and CASP9	• Increases cellular growth, migration, invasion, and proliferation • Increases intracellular ATP, lactate, and pyruvic acid levels	(Kim et al. 2015, Hoffmann et al. 2017, Ptak et al. 2014, Ptak et al. 2011, Hall and Korach 2013)
Endometrial cancer	• Increases expression of EMT markers (VIM, CD90, CD44, CD105), HDAC6 and COX2 through MAPK pathway by estrogenic effect • Downregulates the expression of CDH1, HOXA10, and decidual markers PRL and IGFBP-1 • Activates ERRγ/EGF/EGFR/ERK signaling pathway in Ishikawa cells • Activates the IGF signaling pathway via ERα • Decreases miR-149 expression and downregulates DNA repair gene (ARF6) and p53 and upregulates CCNE2	• Enhances cell proliferation, growth, migration, and invasion • Affects hedgehog signaling via increasing miR-107 expression	(Wang et al. 2015, Klotz et al. 2000, Gertz et al. 2012, Chou et al. 2017, Xiong et al. 2020, Yaguchi 2019)
Cervical cancer	• Activates MMP-2, CDH2, VIM, p65, NF-κB, and IKK-b • Upregulates MMP-9 and Fibronectin	• Induces cell migration and invasion	(Ma et al. 2015)
Prostate cancer	• Stimulates the transcriptional activity of AR-T877A • It activates AR mutant alleles such as T877A, T8775, V715M, L701H, and K580R • Activation of ERK • Downregulation of ERK, cyclin D1, and chromatin-modifying enzymes • Upregulation of p21 and p27 and ion channel protein ORAI1 • Increases aromatase (CYP19A) activity, androgen receptor (AR) expression in the ventral prostate, and also increases centrosome number • Increases DNA methylation and downregulates p16	• Increases cell proliferation, migration, most likely through AR-T877A • Changes cell morphology • Cell cycle arrest • Induction and amplification of calcium entry in LNCaP cells • Alters methylation of tumor suppressor genes • Induces prostate cancer progression	(Wetherill et al. 2006, Bilancio et al. 2017, Derouiche et al. 2013, Fatma Karaman et al. 2019)
Male germ cell cancer	• Activates GPR30, EGFR, ERK, PKG, and AP-1 genes present in the 5′-flanking regions of the GPR30 • Upregulates PKG, ERα, and EGFR/ERK/c-Fos pathways through increased expression of GPER	• • Enhances proliferation of spermatogonial GC-1 cells	(Sheng et al. 2013)
Testicular cancer	• Decreases the testis weight and downregulate the expression of StAR, AMH • Inhibits antioxidant enzyme and elevates lipid peroxidation which in turn enhance oxidative stress in the testis • Increases the number of Leydig cells in adult Long-Evans rats	• Reduces testicular size in male pups • Reduces the daily sperm production, sperm count, fertility and motility • Induces proliferation in testicular seminoma cells through GPER/GPR30	(Xi et al. 2012, Kawai et al. 2003, Chevalier et al. 2012)
Acute myeloid leukemia (AML)	• Activates caspase-3, caspase-8, and caspase-9 • Increases phosphorylation of BAD and acetylation of Histone H3 • Upregulates FAS and TRAIL, IL-6 • Downregulates Cyclin D1, Flip-L, Flip-S, IL-4 • Decrease phosphorylation of ERK, Rb, and AKT	• Induces proliferation and chemoresistance of AML cells • DNA fragmentation • Cell cycle arrest and apoptosis	(Terasaka et al. 2005, Bontempo et al. 2009, S. Zhang et al. 2020)
Lung Cancer	• Activates ERK1/2 through GPER/EGFR and SNAI1-1/CX43/ERRγ-dependent EMT signaling pathway in A549 lung cancer cells • Upregulates GPER, EGFR, ERK1/2, MMP-2, MMP-9,	• Cell migration and invasion • Increases motility of lung adenocarcinoma cells and induces cytoskeleton remodeling • Stimulates invasion in A549 tumor cells through the SNAI1-1/CX43/ERRγ-dependent EMT signaling pathway	(Zhang et al. 2014, Ryszawy et al. 2019)
Colorectal cancer	• Phosphorylates AKT, GSK-3β • Increases expression of SNAIL, TWIST, ZEB and VIM and p38 phosphorylation • Decreases CDH1 expression • Impairs E2-induced extranuclear activities of ERb • Depolarizes MMP and results in loss of mitochondrial integrity	• Increases migration and invasion • Induces toxicity in human colon cancer cells at higher concentration • Causes oxidative damage and increases mitochondrial and intracellular ROS • Increased intracellular release of Ca2+	(Chen et al. 2015, Qu et al. 2018)
Hepatic cancer	• Increases COX1 and G6PC expression while, NUCB2 expression was decreased in female mice • Induces the ACSS2 expression • Elevates SGK1 expression in primary liver cancer	• Induces mitochondrial dysfunction in liver • Alteration in the liver miRNome and transcriptome that causes adverse health effect including cancer • Acts as a partial/competitive agonist for estrogen	(Ilagan et al. 2017, Weinhouse et al. 2014, Moon et al. 2012)
Oral and Oropharyngeal cancer	• Downregulates OPC signaling pathways • Promotes OC and OPC through estrogenic and non-estrogen-dependent pathway	• Involved in the progression of endoderm-derived carcinogenesis	(Li et al. 2017)
Thyroid cancer	• Higher concentrations of urinary BPA were observed in study participants underwent thyroid ultrasonography • Increased concentration of BPA in the blood in patients with thyroid cancer • Upregulation of ER and GPR30 expression in BHP10-3 cells	• High BPA concentration in the body has been associated with an increased risk of thyroid cancer • Enhances thyroid cancer cell proliferation	(Zhou et al. 2017, Li et al. 2019, Zhang et al. 2017)
Osteosarcoma	• Interacts with LOX gene and enhances the risk of the osteosarcoma • Downregulates OPG, RUNX2, and COL1A1 • Inhibits CDC42 expression	• Increases the risk of Osteosarcoma • Changes cell morphology, motility and filopodia formation	(Jia et al. 2013, Fic et al. 2015, Kidani et al. 2017)
Adrenocortical carcinoma	• Stimulates adrenal cell proliferation via ERβ-mediated activation of the Shh pathway • Activates CYP11A1 gene expression and increases corticosterone production through the JNK/c-Jun signaling pathway • Enhances PCNA, cyclin D1 and D2, sonic hedgehog (shh) protein expression	• Stimulates adrenal cell proliferation • Increases adrenal development	(Medwid et al. 2018, Lan et al. 2015)

## Breast cancer

As per the National Cancer Institute and The Institute of Medicine (IOM), BPA is declared as a significant risk factor for breast cancer (BC). Surveillance, epidemiology, and experimental studies have evaluated the association between BPA and BC and demonstrated the potential of low doses of BPA to induce neoplastic lesions. Estrogen, progesterone, and prolactin play an essential role in the development of mammary glands (Rachoń et al. 2008). Endocrine-mediated signaling pathways play a crucial role in the growth, development, and functioning of the mammary gland. EDCs have been reported to alter endocrine-mediated signaling pathways, thus affecting the functions of the mammary gland. Epidemiological reports indicate that elevated levels of estrogen, prolactin, and progesterone are associated with the development of BC (Bernstein 2002; Gao et al. 2015). BPA can be absorbed and stored in human fat tissue and breast stroma, due to its lipophilic nature, and may subsequently activate cancer-promoting signaling pathways to induce BC (Cimmino et al. 2020).

Inappropriate activation of estrogen signaling plays a key role in BC development. Estrogen exerts its effects via nuclear ERα and ERβ or via mERs such as GPER/GPR50 (Prossnitz and Barton 2011). BPA-induced changes in the mammary gland tissues include enhanced estradiol sensitivity and increased progesterone receptors. BPA exposure during mammary gland development is associated with an increased risk of tumorigenesis (Fenton 2006). Low doses of BPA have been shown to stimulate proliferation in both ER-positive and ER-negative cancer cells (LaPensee et al. 2009).

Studies have reported exposing breast cancer cell lines to BPA causes cell proliferation, migration, and invasion (Kim et al. 2017). BPA alters the morphogenesis of the fetal mammary gland through epigenetic modification. Besides, BPA were detected in maternal milk (Mandrup et al. 2016). Exposure to BPA alters DNA methylation and is proposed as a mechanism for increased risk of BC (Fernandez 2012). The study demonstrated that exposure to low doses of BPA induces aberrant methylation of genes such as lysosomal-associated membrane protein 3 (LAMP3). The role of LAMP3 is well established in BC (Weng et al. 2010). BPA promotes migration and invasion of BT-549 and MD-MB-231 cells. Exposure of MCF-7 cell lines to low doses of BPA triggers cell proliferation (Kim et al. 2017). In TNBC cells, the pro-proliferative and pro-survival effects of BPA depend on ERK1/2 and AKT activation (Zhang et al. 2016). Besides, BPA also stimulates the expression of MMPs. The role of ERK1/2 and AKT activation and MMPs is very well established in BC. BPA enhanced migration-related protein and mRNA expression including matrix metalloproteinase-2 (MMP2), and MMP9 is independent of vimentin (VIM) and fibronectin expression in TNBC cells (Zhang et al. 2016).

ERRs, the nuclear receptor superfamily, are also known as orphan receptors because they do not have endogenous ligands. ERRs have estrogen receptor sequence homology but do not bind to estrogen. By binding to ERRγ, BPA stimulates the growth and proliferation of various types of cancer cells including BC. BPA upregulates the EERγ in estrogen receptor-positive (ER^+^) breast cancer cells by phosphorylation of the ERK1/2. These data suggested EERγ/ERK1/2 axis to promote cell proliferation in BC.

Homeobox B9 (*HOXB9*) is activated in response to BPA exposure in BC (Hafezi and Abdel-Rahman 2019). *HOXB9*, located at 17q21.32, participates in cell cycle progression, embryonic patterning, mammary gland development, and cell proliferation. *HOXB9* is upregulated in BC and has been demonstrated to stimulate neovascularization, tumor invasion, and disease progression. The *HOXB9* gene promoter contains a potential estrogen response element (ERE4) which mediates its response to both E2 and BPA (Deb et al. 2016). A study showed that BPA is susceptible to mammotrophic hormones and increases breast cancer risk later in life. This hypothesis was supported by a study that showed the role of BPA in the differentiation of stem cells by altering their sensitivity to BMP signaling, by downregulating the mammary fibroblast development of BMPs, and by altering the localization and expression of type 1 BMP receptors. BPA altered the BMP signaling by SMAD1/5/8 phosphorylation (Clément et al. 2017; Bach et al. 2018). Taken together, various published studies on BC suggest that BPA may impart its pro-carcinogenic effects via inducing epigenetic modifications, DNA damage, stem cell differentiation, and breast microenvironment alteration through activation of pro-carcinogenic signaling pathways.

## Endometrial cancer

Endometrial cancer (EC) is the fourth most common cancer in women reported to originate from a hyperestrogenic pattern (Brooks et al. 2019). Many scientific investigations have proposed endocrine disruptors, including BPA, to be included as a hormonal risk factor category. Due to its estrogen-mimicking properties, BPA is considered a key risk factor for EC. In vitro, in vivo, and human studies indicated BPA as a risk factor of EC. For example, a study by Aquino et al. demonstrated a high concentration of BPA in the urine and blood of EC patients compared to healthy controls. The same study proposes that BPA can directly upregulate the ER genes leading to hyperestrogenism and EC (Aquino et al. 2019). Thus, BPA can favor the hormonal dysregulation at the base of the endometrial neoplasm and contribute to proliferation effects on neogenesis acting on EGF or microRNAs regulation. Overall, estrogen may regulate miRNA transcription through ERα and ERβ in a specific and cell-dependent manner and the fact that EDCs such as BPA would be involved in endometrial carcinogenesis. BPA exposure during critical periods of growth and development can cause an adverse effect at later stages of life. CD-1 female mice exposed to BPA by subcutaneous injection have shown cystic ovaries and endometrial hyperplasia when compared to control mice when examined at 18-month postinjection (Newbold et al. 2007).

The upregulation of cyclooxygenase 2 (COX-2) and EMT pathway genes is related to tumor development and progression. Wang et al. (2015) have reported that 10^-8^ M concentration of BPA enhanced the expression of mesenchymal cell surface markers (*CD44*, *CD90*, *CD105*, and *VIM*) and a cell-cell interaction regulator (HDAC6) and downregulates the expression of epithelial cell-cell adhesion molecule (*CDH1*) in RL95-2 cells. The same study demonstrated that BPA could stimulate the growth, invasion, and migration of RL95-2 cells via the mitogen-activated protein kinase (MAPK) pathway, which possibly leads to the upregulation of COX-2 expression (Wang et al. 2015). BPA elevates ER1 binding sites and alters the expression of a subset of genes affected by E2, leading to the activation of MAPK1, AKT1, and PIK3CA pathways (Gertz et al. 2012). Besides, BPA also activates ERα and ERβ depending in a cell type and concentration-dependent manner. Further, BPA also activates the IGF signaling pathway via ERα in the ovary of adult mice and increases mitotic cells (Klotz et al. 2000; Gertz et al. 2012).

BPA exposure alters microRNA (miRNA) expression to contribute to EC. Chou et al. carried out a transcriptomic analysis to discover altered mRNA and miRNAs in response to BPA exposure in human endometrial cells. The results showed that exposure to BPA decreases the expression of miR-149, downregulates DNA repair gene (*ARF6*) and *TP53*, upregulates *CCNE2*, impairs the cell cycle, and initiates cell migration and invasion. Further, BPA affects hedgehog signaling via an increase in miR-107 expression (Chou et al. 2017).

A recent study that determined the effect of BPA on endometrial stromal cell decidualization suggested the potential crosstalk between BPA and epigenetic modifications. The study elaborates on the crosstalk between BPA and histone modifications during endometrial stromal cell decidualization resulting in the downregulation of *HOXA10*, *PRL*, and *IGFBP-1* (Xiong et al. 2020). BPA exposure induces the proliferation of HEC265 cells and Ishikawa cells by nuclear translocation of ERRγ and increased BPA/ERRγ-target gene expression. In addition, BPA facilitated the Ca^2+^ influx in Ishikawa cells and EGF secretion to the extracellular space, activating the EGFR/ERK pathway. BPA enhanced the expression of BPA/ERγ-target genes in HEC265 cells, without affecting Ca^2+^ mobilization and EGF secretion (Yaguchi 2019). These data suggest that BPA might activate multiple signaling pathways to stimulate the proliferation of EC cells. However, more detailed mechanistic studies are needed to understand the impact of BPA in EC.

## Ovarian cancer

Ovarian cancer (OVC) ranks the seventh most common cause of cancer mortality among women with the worst prognosis and high mortality rate (https://gco.iarc.fr/). In addition to family history, the risk factors for OVC include obesity, smoking, alcoholism, late menopause, hormone replacement therapy, early menarche, nulliparity, and mutations in *BRCA1/2* (Brett et al. 2017; Jammal et al. 2017). Besides this, exposure to EDCs is reported as one of the significant risk factors for OVC (Rachoń 2015). Sex steroids play an important role in OVC development and progression (Gómora et al. 2018). There is an elevated level of ER expression in epithelial ovarian cancer cells compared to benign and normal ovarian epithelial cells (Ajani et al. 2017). Estrogen consumption, either as an oral contraceptive or hormone replacement therapy, increases the risk of OVC. Estrogen is reported to facilitate tumor progression via activation of pro-proliferative and pro-survival cellular mechanisms. BPA mimics estrogen. Hence, BPA exposure may mimic the effects of estrogen to promote OVC. Based on the epidemiological data and molecular investigation, BPA is proposed as a potential risk factor in OVC.

BPA is reported to interrupt steroidogenesis in the ovary, facilitating the development of polycystic ovary syndrome (Konieczna et al. 2018). Various studies in the rodent model suggested that BPA exposure induces morphological alterations in the ovary, increase in the number of atretic follicles and farm cystic ovaries, endometrial hyperplasia, reduced primordial follicles, and other problems associated with ovary development (Pivonello et al. 2020). Exposure to BPA was reported to induce gene expression, some of which are activators of oncogenic signaling. Both estrogenic-dependent and estrogenic-independent signaling mechanisms are proposed for BPA in ovarian tissues. BPA promotes proliferation, invasion, angiogenesis, and chemoresistance in ovarian cancer via phosphorylation of signal transducer and activator of transcription 3 (*STAT3*), extracellular signal-regulated kinases (ERK1/2), and activation of *CDH2* (N-cadherin), *MMP2*, and *MMP9* leading to the induction of epithelial-mesenchymal transition (EMT) (Ptak et al. 2014; Hafezi and Abdel-Rahman 2019). Derailed expression of *CDH1* (E-cadherin) is implicated in the invasion and metastasis of OVC. BPA downregulates *CDH1* expression in ovarian tumors (Ptak et al. 2014). Likewise, the presence of BPA in OVC cells has been shown to increase *SNAI1* response via ER-α and ER-β (Kim et al. 2015). The same study showed the impact of BPA on OVCAR-3 cell migration compared to 17β estradiol (E2). Ptak et al. (2014) have reported that elevated BPA level induces matrix metalloproteinase-2 *(MMP-2)* and *MMP-9* and *CDH2* in turn facilitates cell migration (Ptak et al. 2014). Studies have reported that treatment with BPA can upregulate cyclin-dependent kinase 4 *(CDK4)*, cyclin E1 *(CCNE1)*, cyclin D1 *(CCND1)*, insulin-like growth factor 1 receptor *(IGF-1R)*, B cell lymphoma 2 *(BCL2)*, and ER-α and downregulate aryl hydrocarbon receptor nuclear translocator 2 (*ARNT2)* and *CDKN1A*, resulting in cell proliferation and apoptosis inhibition (Ptak et al. 2011). BPA cooperates with leptin to inhibit caspase-3 expression and OVC cell function. BPA was shown to increase the expression of leptin receptors and to induce proliferation by STAT3, ERK1/2, and AKT phosphorylation (Ptak and Gregoraszczuk 2012). Interestingly, Hoffmann et al. showed that low concentrations of BPA induced OVC progression by upregulation of Apelin (APLN) expression through peroxisome proliferator-activated receptor gamma (PPARγ) (Hoffmann et al. 2017). *APLN* is an endogenous ligand for G protein-coupled APJ receptors. The *APLN* gene is enhanced by tumor necrosis factor-α (TNF-α). Interestingly, reports also show that low exposure of BPA leads to increased cell viability, cell proliferation, and glycolysis, resulting in elevated levels of intracellular ATP, lactate, and pyruvic acid leading to augmented proliferation in OVCAR-3 cells via ERα pathway (Shi et al. 2017). Exposure of SKOV3 cells to low doses of BPA induced EMT via canonical Wnt pathway activation. The same study has shown that low dose of BPA exposure significantly induced migration without altering cell proliferation in SKOV3 and A2780 cells (Lin et al. 2018). BPA is an activator of phosphatidylinositol-3-kinase (PI3K) signaling in OVC. *SnoN* is a negative regulator of TGF-β signaling and an activator of the tumor suppressor p53 in response to cellular stress. BPA promotes the growth and proliferation of OVC cells by upregulating *SnoN* and *APLN* expression (Park and Choi 2014). Caspases are required for completing apoptosis, and caspase-9 and caspase-3 are involved in apoptosis induction and execution, respectively. By downregulation of caspase-3 and caspase-9, BPA inhibits apoptosis in OVC cells. Transplacental exposure to BPA induced changes in the expression of genes linked to estrogenic activity in Sprague-Dawley rat ovaries (Naciff 2003). Furthermore, BPA regulated the expression of BCL2-associated X, apoptosis regulator *(BAX)*, *BCL2*, *CDK4*, and *CCNE1* through the genomic estrogenic pathway. *BCL-2/BAX* plays an important role in regulating caspase-dependent and caspase-independent apoptosis. *CDK4* plays an essential role in the G1 to S phase of the cell cycle (Ptak et al. 2011; Peretz et al. 2012; Gao et al. 2015). In OVC, BPA stimulates ERα expression and enhances the growth of OVC cells by activating ER and augmenting the CXC motif, chemokine 12 (CXCL12)- CXC chemokine receptor 4 (CXCR4) signaling axis (Hall and Korach 2013). Chemokine receptor CXCR4 and its ligand CXCL12 play an essential role in the metastatic homing of tumor cells.

BPA exposure resulted in an elevated level of SnoN expression and decreased phosphorylation of Smad3 in BG-1 cells via inhibition of the TGF-β signaling pathway. Likewise, transplanted ovarian BG-1 cancer cells in the xenograft mouse model showed increased expression of SnoN and reduced phosphorylation of SMAD3 after treatment with BPA (Park and Choi 2014). Despite the evidence which shows that BPA exposure can affect ovarian function and can stimulate the oncogenic signaling pathway in OVC cell lines, there is still a lack of epidemiological data to support BPA’s role in OVC incidence. Hence, more detailed epidemiological functional studies are required.

## Cervical cancer

Cervical cancer (CC) is not an estrogen-dependent cancer. However, various experimental and epidemiology data showed that exposure to EDCs is a potential risk factor for CC. The association between diethylstilbestrol and vaginal clear cell adenocarcinoma was the first report linking EDCs and cancer (Reed and Fenton 2013; Tournaire et al. 2015). Subsequently, studies have shown diethylstilbestrol as a risk factor for CC. A study performed by Ma et al. demonstrated BPA as a promoter of migration and invasion of SiHa, HeLa, and C-33A cells via modulation of estrogen signaling (Ma et al. 2015). Nevertheless, Den et al. demonstrated the role of stromal estrogen signaling in CC development (den Boon et al. 2015). Interestingly, cervical tissue is hormone-sensitive, and it shows a high response in the presence of estrogen. Thus, the pro-migratory and pro-invasive response to BPA exposure may be due to the hormone-sensitive nature of cervical tissue. Studies showed that the BPA level in the human body corresponds with the occurrence of cervical CC. High BPA levels are detected in urine samples of CC patients (Ma et al. 2015). BPA activates nuclear factor kappa B (NF-κB) and upregulates MMP-9 and fibronectin (FN) in SiHa and Hela cells. Upon BPA exposure, IKKβ activates NF-κB signaling leading to its nuclear translocation and results in cell migration and upregulation of FN and MMP-9. Thus, in CC, NF-κB, FN, and MMPs are the target genes of BPA (Ma et al. 2015). BPA also enhances the incidence of proliferative lesions of the uterus as well as sarcoma of the uterine cervix (Pivonello et al. 2020). A study by Newbold et al. reported that BPA exposure enhances the incidence of stromal sarcoma of the cervix (Newbold et al. 2009). Although BPA is linked to CC, its precise role, exact molecular mechanism, biological function, and signaling pathways regulated by BPA in CC are yet to be identified and established. Hence, further epidemiological and functional studies are required.

## Prostate cancer

Prostate cancer (PC) is the cancer of the male reproductive tissue. Epidemiology and experimental studies have demonstrated the role of BPA as a risk factor for PC. Besides, in vitro and in vivo models have shown that BPA can influence the progression of PC. Early-life exposure to BPA increases the susceptibility to hormone-related carcinogenesis in the prostate gland (Prins et al. 2015). Various preclinical model-based studies demonstrated the role of estradiol levels in PC pathogenesis. Reports have suggested an increase in the level of estradiol during aging and related studies conducted in an animal models have shown BPA exposure can lead to an increase in estradiol levels in aging male rats, contributing to PC’s susceptibility (Prins et al. 2017). Many studies have also reported BPA exposure to induce an abnormal epigenetic modification in genes belonging to multiple signaling pathways (Ho et al. 2006). Defective calcium signaling is associated with cell migration in different cancer types including PC. Besides, BPA also activates key genes linked to proliferation and cell survival. For instance, Prins et al. showed that BPA enhanced the phosphorylation of AKT and ERK in human stem cells expressing ER and GPR30 in dose-dependent manner (Prins et al. 2014).

Defective calcium signaling is associated with the cell migration in different cancer types, including PC. BPA is reported to enhance the migration of PC cells via modulating calcium signaling. For example, Derouiche et al. (2013) have shown that BPA modulates calcium signaling to promote the migration of LNCaP cells. Treatment of LNCaP cells with 1 and 10 nM of BPA showed a significant increase in cell migration. The same study also showed that BPA induces PC cell migration via modulating the expression of an ion channel protein associated with calcium entry, namely, ORAI1 (Derouiche et al. 2013). Very interestingly, in mice, the treatment resulted in high levels of AR and ER when compared with the respective controls. This suggests that BPA exposure may lead to inappropriate stimulation of AR and ER activation pathways (Di Donato et al. 2017). The centrosome plays an important role in cell cycle regulation and is currently being recognized as a key target for cancer therapy. Centrosome aberration is highly prevalent in cancer as it affects cell cycle progression. Exposure to BPA has been reported to enhance the centrosome number in both normal and PC cells (Tarapore et al. 2014). Besides, low doses of BPA disturb the centrosome duplication cycle. Bilancio and co-workers demonstrated BPA-induced cell cycle arrest in both prostate cancer (LNCaP) and normal prostate epithelial (EPN) cells. The treatment of LNCaP and EPN with 10 to 100 μM of BPA induced cell cycle arrest by lowering *CCND1* and concomitant upregulation of p21 and p27. Besides, the same study also showed the activation of EGFR- and ERK-dependent pathways (Bilancio et al. 2017).

BPA induces a variety of epigenetic modifications in a wide array of cells. Studies on PC cells have suggested that BPA induces posttranscription and posttranslational modifications, both globally and regionally. Thus, BPA-induced epigenetic modification may contribute to the abnormal biological behavior of the cell. To unravel this, a research examined the impact of BPA on the expression of chromatin-modifying enzymes, promoter methylation of tumor suppressor genes, and histone modifications in PC-3 cells. Treatment with BPA induced hypermethylation of the p16 promoter, leading to its downregulation. The same study reported significant changes in global histone modifications (*H3K9ac*, *H3K9me3*, *H3K27me3*, and *H4K20me3*) in PC-3 cells possibly via downregulation of chromatin-modifying enzymes including nuclear receptor binding SET domain protein 1 *(NSD1)* and lysine demethylase 5B *(KDM5B)* and altered promoter methylation of tumor suppressor genes (*BCR*, *GSTP1*, *LOX*, *MGMT*, *NEUROG1*, *PDLIM4*, *PTGS2*, *PYCARD*, *TIMP3*, *TSC2*, and *ZMYDN10*). The ChIP results showed a significant increase (1 and 10 μM of BPA) in histone modifications (Fatma Karaman et al. 2019). Neonatal or early-life exposure to BPA is a risk factor for PC later in life. This could be because of the developmental reprogramming of the prostate gland induced by epigenetic reprogramming. A study by Prins and co-workers using rat models proposed that BPA exposure can increase PC susceptibility to epigenetic modification through the induction of global hypomethylation of key cancer susceptibility genes (Prins et al. 2017). Overall, BPA is a potent regulator of epigenetic enzymes and can bring about abnormal epigenetic changes in PC and may facilitate its progression.

## Testicular germ cell cancer

Male germ cell cancer represents nearly 2% of all cancers at risk. Occupational exposure to BPA is proposed as a potential risk factor for male germ cell cancer. In vitro studies have demonstrated that BPA can alter proteins and affect the functional characteristics of the germ cells of the testis and may affect male fertility. Reports indicated an exposure of male germ cells (spermatogonial GC-1 cells) to 1000-μM BPA for 48 h activates various genes such as GPR30, EGFR, ERK, and PKG. Low doses of BPA boost the proliferation of spermatogonial GC-1 cells via EFGR/ERK/GPR30/c-Fos/ER-α/PKG axis (Nomiri et al. 2019). Male germ cell tumors show overexpression of GPR30. Similar to ERs, GPR30 controls the cellular response towards 17β-estradiol. Besides, GPR30 is proposed as a molecular target in male germ cell cancer. Sheng et al. (2013) showed that a low dose of BPA induces the progression of male germ cell cancer and activates the expression of GPR-30 via EFGR/ERK/GPR30/c-Fos/ER-α/PKG pathway (Sheng et al. 2013). Fetal exposure to BPA has an adverse effect on the male reproductive system. BPA is known to affect the quality and quantity of sperms. Gestational exposure of female mice to BPA results in a decrease in testis weight and size, downregulation of anti-Mullerian hormone (AMH), and steroidogenic acute regulatory protein (StAR) in male pups (Kawai et al. 2003; Xi et al. 2012). Many studies have established a relationship between environmental pollutants, impairment in male germ cell development, and testicular tumor development. As per the study by Delbès et al., BPA exposure resulted in the reduction of sperm production and enhanced the incidence of testicular cancer (TC) (Delbès et al. 2006). BPA increases seminoma cells proliferation by activating GPR30 (Chevalier et al. 2012). Besides, fetal exposure to BPA has been reported to induce reproductive dysfunction and contribute to male infertility and TC (Adiga et al. 2020). The low-dose BPA exposure induced seminiferous tubule cell proliferation in a PKA-PKG-GPER–dependent manner (Cariati et al. 2019). Compromised immunity plays an important role in cancer development and progression. A study by Nava-Castro et al. investigated the role of BPA on immune response and TC. The same study showed that exposure to BPA in pregnant female mice increases testicular tumor size via effecting the immune component and immune response in male offspring’s (Nava-Castro et al. 2019). Overall, studies show that BPA can affect the male reproductive system, induce reproductive dysfunction, and may contribute to testicular germ cell cancer. However, further detailed investigations are required before conclusions are drawn.

## Acute myeloid leukemia

Acute myeloid leukemia (AML) is a hematopoietic stem cell malignancy. The estrogenic signals can play a key role in the progression of AML. Studies have reported the presence of BPA in the serum sample of AML patients (Zhang et al. 2020). Both cell proliferation and apoptosis induction properties are reported for BPA (Bontempo et al. 2009). One of the earliest studies has shown the cell cycle arrest and apoptosis-inducing properties of BPA in AML cells treated with a micromolar concentration of BPA (Terasaka et al. 2005). BPA treatment induced apoptosis in NB4 cells via activation of caspases involving FAS/TRAIL/BAD axis (Bontempo et al. 2009). It induces inter-nucleosomal DNA fragmentation and activates caspase-9 and caspase-3, implicating the induction of apoptosis (Terasaka et al. 2005). In contrast to this study, another study demonstrated BPA to induce cell proliferation and resistance to daunorubicin and cytarabine in AML (Zhang et al. 2020). Daunorubicin and cytarabine are chemotherapeutic drugs. Specifically, it is used for the treatment of AML. Abnormal expression of cytokines and chemokines plays an important role in AML progression. BPA-induced cell proliferation involves the upregulation of interleukin 6 (*IL6*) and downregulation of *IL4* (Zhang et al. 2020). Activation of NF-κB upregulates *IL6* while NFAT contributes to *IL4* downregulation. More recent studies report BPA to trigger AML (Terasaka et al. 2005).

## Lung cancer

The endocrine-disrupting property of BPA is reported to enhance the susceptibility to lung cancer (LC). Accumulating evidences have suggested that estrogen and its receptors can contribute to LC. The lung alveolar cells get exposed to BPA via inhalation and may alter the functional properties of the cells. A Chinese population-based case-control study demonstrated that BPA levels were significantly higher in non-small cell lung cancer cases compared to control samples (Li et al. 2020). Zhang and colleagues demonstrated the role of BPA in altering the biological behavior and function of lung cancer cells. BPA concentration less than 10^-4^ M enhances the migration and invasion of A549 cells (Zhang et al. 2014). Besides this, BPA also upregulated the expression of MMP2 and MMP9 by ERK1 activation through GPER/EGFR (Zhang et al. 2014). ERK1 and MMPs activation are potential pro-carcinogenic signals in numerous cancers, including LC. Similarly, another study involving the cross talk between BPA and induction of pro-metastatic signaling suggested the activation of *SNAI*1/Cx43/ERRγ-dependent EMT signaling pathway upon BPA exposure in A549 cells (Ryszawy et al. 2019). BPA induces morphological changes and enhances motility by cytoskeletal rearrangements (Zhang et al. 2014). Some studies reported BPA to induce EMT in lung cancer via a switch from *CDH1* to *CDH2* and vimentin/*SNAI*1/connexin (Cx) 43 upregulation (Ryszawy et al. 2019). Besides inducing migration and invasion, BPA exposure is responsible for the inflammation and oxidative stress in rat lung tissues. The same study showed that BPA upregulated malondialdehyde and IL18 with a reduction in superoxide dismutase (SOD) levels. Oxidative stress and inflammatory pathways in lung cells are promoters of LCs (Abedelhaffez et al. 2017).

## Colorectal cancer

Studies have shown that estrogens and their structural analogous along with their receptors are involved in intestinal diseases and development and progression of colorectal cancer. E2-induced caspase activation is essential for apoptosis in many cell types. Apoptosis induced by activation of E2 is impaired in colon cancer cells. Chen and co-workers in 2014 investigated the effect of BPA on colorectal cancer using a proteomic approach. In SW80 cells, BPA treatment altered the expression of 56 proteins related to structure, movement, proliferation, and others. Besides, they showed EMT, spindle-shaped mesenchymal morphology with upregulation of *CDH2* and *SNAI1*. *CDH2* is a transmembrane protein, and it functions to mediate cell-cell adhesion, and it is a hallmark of EMT. *SNAI1* is a typical transcription factor that could induce EMT and cancer progression with concomitant downregulation of *CDH1* (Chen et al. 2015). This suggests that, in colon cancer, BPA may participate in the induction of aggressive phenotypes. The BPA effect on colon cancer is diverse. Glycogen synthase kinase 3β (GSK3β) is a multifunctional protein involved in various cellular activities such as development, differentiation, and disease. By altering GSK3β expression, BPA promotes migration and invasion of colon cancer cells via activating of BCL-xl and inhibits apoptosis (Chen et al. 2015). A recent study showed that 250 μM of BPA has induced toxicity in human colorectal cancer cells (Qu et al. 2018). It has been reported that BPA causes oxidative damage to the colonic epithelium as shown by increased mitochondrial and intracellular ROS and increased levels of hydrogen peroxide and malondialdehyde (Wang et al. 2019). BPA increased the intracellular release of Ca^2+^ and is responsible for the depolarization of MMPs and the loss of mitochondrial integrity in HCT116 cell lines (Qu et al. 2018).

## Hepatic cancer

Hepatic tissue is the nonreproductive target of estrogen. Many environmental agents are reported to cause hepatocellular carcinoma (HCC). The primary risk factor for HCC is chronic hepatitis B and C virus infection, environmental exposure, and excessive alcohol consumption (Balogh et al. 2016). The liver expresses ERs and responds to signaling related to steroid hormones. BPA is demonstrated to cause hepatic toxicity and liver injury (Hassan et al. 2012; Thoene et al. 2017). BPA can induce liver damage by oxidative stress (Esplugas et al. 2018). Studies have demonstrated that ~70% of the fetal liver in humans shows the presence of detectable BPA levels and is linked to altering the methylation levels of xenobiotic-metabolizing enzymes. The first systematic study, conducted by Weinhouse et al*.*, demonstrated the potential role of an environmentally relevant dose of BPA in inducing hepatic tumors in AvyC3HeJ/C57BL/6 mice (Weinhouse et al. 2014) BPA exposure also induced both tumor and precancerous conditions. Mitochondrial dysfunction is potentially related to carcinogenesis. Many previous studies have clearly demonstrated that epigenome-wide changes, oxidative stress, inflammation, and mitochondrial damage may lead to liver damage and subsequently to HCC later in life. BPA induces mitochondrial dysfunction and hepatic injury by enhancing oxidative stress in the liver (Moon et al. 2012). BPA is reported to alter mitochondrial structure, increase malondialdehyde levels, and decrease glutathione peroxidase 3 expression (Moon et al. 2012). The increase in *IL6* and TNF-α levels was reported, suggesting that intraperitoneal administration of BPA can induce inflammation of liver cells in mice (Moon et al. 2012). In HepG2 cells, nanomolar concentration of BPA has shown to reduce oxygen consumption rate, ATP level, and mitochondrial membrane potential (Huc et al. 2012). Studies conducted in a zebrafish models have revealed that BPA can induce hepatic epigenetic alterations. The same study has also reported altered expression of genes related to mitochondrial functions including oxidative phosphorylation, in response to BPA exposure (Renaud et al. 2017). BPA enhances the expression of *COX1* and *G6PC* genes in female mice compared to male mice, while nucleobindin-2 (NUCB2) expression was decreased in female mice. BPA induces *ACSS2* expression, which is a cancer susceptibility gene. Serum/glucocorticoid-regulated kinase 1 expression is elevated in primary liver cancer (Ilagan et al. 2017). BPA affects liver functions that are evident by the reduction in the activities of several enzymes, including catalase, glutathione, and others (Aboul Ezz et al. 2015). BPA is reported to cause liver injury, which included necrosis, vacuolization of the cytoplasm, and decreased hepatocellular compactness (Sangai et al. 2014). The epigenome-wide changes, oxidative stress, inflammation, and mitochondrial damage may lead to liver damage and subsequently to HCC later in life.

## Head and neck cancer

Oral cancer (OC) and oropharyngeal cancer (OPC) are one of the groups of head and neck cancer. The risk factor for OC includes the use of tobacco products, excess alcohol intake, and diet. Oral cavity and oropharyngeal space are the first sites of exposure for ingested environmental toxicants. Thus, the oral cavity and oropharyngeal space are at high risk for BPA-induced carcinogenesis. BPA can promote OC and OPC through the estrogenic- and non-estrogen-dependent pathway. Li and colleagues reported that BPA facilitates proliferation, migration, and invasion of laryngeal squamous cell carcinoma (LSCC). There was an upregulation of MMP2 and *IL6*, suggesting the activation of inflammatory pathways by BPA. Further, the proliferation and migration induced by BPA is a GPER- and IL-6-dependent process (Li et al. 2017). The current literature survey suggests a lack of data on BPA and head and neck cancer. Hence, more detailed functional studies are needed to assess the toxic effects of BPA on OC and OPC.

## Thyroid cancer (TCs)

EDCs have emerged as a major public health problem globally. EDCs have been reported to modify the natural endocrine function as they can directly interact with steroid hormone receptors. One of the earliest studies demonstrated that BPA, by acting as an antagonist, disrupts the action of thyroid hormone. Several investigators have evaluated the relationship between EDCs and TCs. Being a member of EDC, BPA may affect the thyroid hormone and its action. Li et al. reported the higher incidence of thyroid nodules in Chinese women who are exposed to BPA (Li et al. 2019). A study by Lee and co-workers demonstrated the positive correlation between the BPA levels in blood with that of ANXA6 and valosin-containing protein expression in TC patients (Lee et al. 2018). The relationship between higher urinary BPA levels along with higher iodine intake was linked to papillary thyroid carcinoma (Zhou et al. 2017). Another case-control study demonstrated the association between higher concentrations of urinary BPA with an increased risk of TC (Li et al. 2019). BPA treatment enhances the H_2_O_2_ generation in PCCL3 cells and suggests that BPA may promote oxidative stress and damage thyrocytes leading to thyroid disorders (da Silva et al. 2018). BPA enhances thyroid cancer cell proliferation through the regulation of ER and GPR30 expression (Zhang et al. 2017). Taken together, there may be a connection between BPA and thyroid nodules or cancer, but further investigations are required to correlate BPA and thyroid carcinogenesis.

## Osteosarcoma

Environmental BPA accounts for the pathogenesis of osteosarcoma. In vitro and in vivo studies have shown that exposure to BPA is associated with the occurrence of osteoporosis. A Chinese hospital-based case-control study has reported that BPA exposure induces a genetic variation −22G/C polymorphism of the lysyl oxidase gene (*LOX*) and enhances the risk of osteosarcoma (Jia et al. 2013). BPA downregulate the osteoprotegerin (*OPG)*, runt-related transcription factor 2 (*Runx2)*, and protein-coding collagen type I alpha 1 *(COL1A1)* genes in HOS cells (Fic et al. 2015). OPG and Runx2 are the major transcription factors playing an important role in adult bone remodeling. *COL1A1* is a bone matrix protein gene; it is regulated by Runx2. BPA exposure increased the plasma levels of procollagen type I N-terminal propeptide (P1NP) associated with the risk of bone metastasis (Lind et al. 2019). Further studies are needed to understand further the molecular mechanisms of BPA in osteosarcoma.

## BPA and therapeutic resistance

Therapeutic resistance is a major problem in cancer therapy and contributes to a high mortality rate. Metastatic cancer patients develop chemoresistance to several drugs. Several in vitro studies showed that BPA exposure could promote therapy-resistant phenotypes in BC. Several reports have demonstrated that anticancer drugs have been shown to be antagonistic to estrogen. BPA has a high binding affinity towards that of estrogen-related receptor-γ (ERRγ). BC patients have shown resistance to tamoxifen, lapatinib, doxorubicin, and cisplatin; BPA have been shown to inhibit the efficacy of doxorubicin by enhancing the levels of BCL-2 and BCL-xL (Barret 2009). The study confers that, in both ERα-positive and ERα-negative BC cells, BPA antagonizes the cytotoxic effect of chemotherapy agents such as doxorubicin, cisplatin, or vinblastine. The same study also reported that BPA exhibits its anticytotoxic function by inhibiting ERα or ERβ, suggesting the potential role of BPA to activate nonclassical ER(s) (LaPensee et al. 2009). Both MDA-MB-468 and T47D cells expressing GPR30, ERRα, and ERRγ showed increased anti-apoptotic BCL-2 protein expression upon treatment with doxorubicin and BPA (LaPensee et al. 2009). BPA-mediated anticytotoxicity in cancer cells also involves EGFR signaling pathway (Sauer et al. 2017). Studies have shown the role of BPA activating the EGFR/ERK1/2 pathway, leading to increased expression of an anti-apoptotic protein (Hafezi and Abdel-Rahman 2019). Tamoxifen (TAM) is an ER modulator selective for treating ER^+^ breast cancer. In vitro research has shown that BPA neutralizes the tamoxifen effect by avoiding apoptosis induced by TAM (Hafezi and Abdel-Rahman 2019). BPA to inhibit rapamycin’s pro-apoptotic effects is also reported (Dairkee et al. 2013). BPA has consistently induced activation of the mammalian rapamycin (mTOR) pathway, followed by dose-dependent evasion of apoptosis and increased proliferation of HRBECs by downregulating the tumor protein 53 (TP53), p21, and *BAX* pro-apoptotic proteins with concomitant increases in gene products that cause proliferation (Dairkee et al. 2013).

BPA exposure upregulates the eukaryotic initiation factor 4A-1 (TIF4A) and fascin, and it also downregulates cytokeratin (KRT8) in colon cancer cells. Overexpression of fascin is associated with the poor prognosis in colorectal cancer cells and it induces doxorubicin resistance in xenografted BC cells by activating the PI3K/AKT signaling pathways (which are involved in survival and proliferation) and inhibition of pro-apoptotic genes, namely, caspase-9 and caspase-3 (Ghebeh et al. 2014; Chen et al. 2015). *HOXB9* is another gene whose expression was induced after exposure to BPA in many cancer cells. *HOXB9* activation is reported to induce chemoresistance to anti-VEGF bevacizumab in colorectal cancer xenograft mouse models via altering angiogenic factors expression such as CXCL1, TGF-β1, angiopoietin-like 2 (Angptl2), and IL8 (Carbone et al. 2017). BPA has altered the development of various types of collagen through various cells associated with drug resistance. In vitro studies reported that A2780 ovarian cancer cells are resistant to the chemotherapeutic drug cisplatin when cultured on collagen (Januchowski et al. 2016; Hafezi and Abdel-Rahman 2019). BPA exposure in PC has been reported to play a role in resistance to androgen deprivation therapy, which blocks androgen receptor (AR) activity. A study by Wetherill et al. demonstrated that BPA induce proliferation of LNCaP cells via activation of AR-T877A receptor, which may contribute to androgen deprivation therapy resistance in a subset of prostate tumor cells (Wetherill et al. 2006). BPA activates the androgen receptor mutant AR-T877A, which results in the dimerization of the mutant AR and its dissociation from the heat shock protein and localization to the nucleus and upregulation of target genes (PSA). BPA also reduces the regulation of ERβ (Hafezi and Abdel-Rahman 2019). Altogether, various studies have shown that BPA can induce chemotherapeutic resistance in a variety of cancer types (Table 2).

**Table 2:** BPA association with therapeutic resistance and disease outcome in cancer

Cancer	Drug resistance or therapeutic resistance	Mechanism of action	Reference
Breast Cancer	Doxorubicin	BPA along with doxorubicin increases expression of anti-apoptotic proteins such as Bcl-2 and Bcl-xL	(Barret 2009)
	Doxorubicin, cisplatin, or vinblastine	BPA exerts its anticytotoxic activity while inhibiting ERα or ERβ	(LaPensee et al. 2009)
	Lapatinib	Inhibits EGFR/ERK1/2 pathway and increases anti-apoptotic protein levels	(Sauer et al. 2017)
	Tamoxifen (TAM)	BPA neutralizes the effect of tamoxifen; it bypasses TAM induced apoptosis	(Hafezi and Abdel-Rahman 2019)
	Rapamycin	BPA inhibits rapamycin’s pro-apoptotic effects; it activates mTOR pathway, evasion of apoptosis by downregulating the p53, p21, and BAX proteins	(Dairkee et al. 2013)
Ovarian Cancer	Cisplatin	BPA induces collagen overexpression; the resistance to chemotherapeutic drug cisplatin by inhibiting cisplatin’s apoptotic property	(Januchowski et al. 2016; Hafezi and Abdel-Rahman 2019)
Prostatic Cancer	Androgen deprivation therapy (ADT)	BPA activates the androgen receptor mutant AR-T877A; which leads to the dimerization of mutant AR and its dissociation from heat shock protein; nucleus location and target gene (PSA) upregulation and BPA also downregulates the ERβ	(Hafezi and Abdel-Rahman 2019)
Colorectal Cancer	Doxorubicin	BPA upregulate fascin expression that cause resistance to doxorubicin by activating PI3K / AKT pathway, inhibits apoptosis by suppressing pro-apoptotic caspase-9 and caspase-3	(Ghebeh et al. 2014, Chen et al. 2015)
	anti-VEGF bevacizumab	BPA induced expression of HOXB9 gene; HOXB9 induces chemoresistance to the anti-VEGF bevacizumab by regulating angiopoietin-like 2 (Angptl2), CXCL1, TGF-β1, and IL8 angiogenic expression	(Carbone et al. 2017)
Acute myeloid leukemia	Daunorubicin and cytarabine	BPA decreases the expression of interleukin-4 (IL-4) while increasing the expression of IL-6. Activates NF-κB, enhances NFAT1 expression and decreases daunorubicin and cytarabine therapeutic effect	(Zhang et al. 2020)
Renal cell carcinoma	Irinotecan	Imparts noncompetitive inhibition on the activity of UGT1A1 and inhibits the function of irinotecan	(Jiang et al. 2016)

## CLARITY-BPA study

The National Center for Toxicological Research (NCTR) of the U.S. Food and Drug Administration (FDA), the National Toxicology Program (NTP), and the National Institute for Environmental Health Sciences (NIEHS) have conducted a Consortium Linking Academic and Regulatory Insights on Toxicity of BPA (CLARITY-BPA) to provide more clarity on toxicological and potential health effects of BPA exposure using Sprague-Dawley rats in compliance with Good Laboratory Practice (GLP). Camacho et al. 2019 showed that BPA exposure did not show any BPA-related health effects in NCTR Sprague-Dawley rats (Camacho et al. 2019). However, rats exposed to 25,000 μg BPA/kg bw/day BPA showed a possible relationship between increases in the incidence of lesions in the reproductive tract of females and pituitary males (Camacho et al. 2019). Another CLARITY-BPA study investigated the adverse effect of chronic exposure to BPA on the immune system (Li et al. 2018). There was no significant change observed in the immune cell composition after chronic exposure to BPA (Li et al. 2018). A study by Prins and co-workers in 2018 using NCTR, Sprague-Dawley cesarean-derived rats, and prostate epithelial cells proposed that BPA exposure can increase the risk of aging-associated cancers such as PC (Prins et al. 2018). To have better clarity on the CLARITY-BPA studies’ findings, Heindel and co-workers consolidated the findings of various studies (Heindel et al. 2020). The same report proposes that BPA exposure during development can adversely affect multiple organs’ functioning (Heindel et al. 2020). Besides, as per the CLARITY-BPA core study, BPA at a dose of 2.5 μg/kg/day induced adenocarcinoma of the mammary gland. Another CLARITY-BPA consortium study reported the induction of epigenetic alterations in the hypothalamus and hippocampus of rats exposed to BPA (Cheong et al. 2018). Besides, BPA exposure resulted in a significant increase in ovarian follicular cysts and mammary adenocarcinoma in female Sprague-Dawley rats. Taken together, the CLARITY-BPA confirms previous findings, which suggests that BPA exposure affects multiple organ systems. Besides, low-dose exposure can increase prostate cancer risk or susceptibility. Also, developmental exposure to 2.5 μg/kg BW/d increases mammary and prostate cancer risk.

## Conclusion

BPA is majorly used in the manufacture of plastic and epoxy resins. In vitro and in vivo studies have suggested BPA with potential carcinogenic functions. Studies using various model systems have suggested BPA exposure can significantly impact growth, survival, proliferation, invasion, migration, and apoptosis in a variety of cell types, including cancer cells. Besides, exposure to BPA may also facilitate chemotherapy resistance to anticancer drugs such as cisplatin, tamoxifen (TAM), doxorubicin, carboplatin, bevacizumab, irinotecan, PARP inhibitors, vinblastine, and other drugs. BPA interacts with several receptors and causes aberrant changes in several pathways such as JAK/STAT, MAPK/ERK, P13K/AKT, c-RAF, HER3, BCL-2, PR-A, SRC1-3, PCNA, PTEN, phosphorylation IRS, cyclin D1, AKT, PPARγ, and *APLN*. It upregulates the mRNA levels of ERα, IGF-1R, EGFR, GPER, ERK1/2, c-Fos, EGR-1, and CTGF genes. Although several studies have suggested BPA’s potential role in carcinogenesis, there are also studies reporting no carcinogenetic role to BPA. Although in vitro and in vivo studies have suggested a possible pro-carcinogenic role to BPA, however, still, there is inadequate epidemiological evidence to consider BPA as a human carcinogen. Therefore, there is a need for some more comprehensive studies to unravel the effect of BPA at a molecular level in various cancers. Understanding the potential impact of BPA on cancer may help raise awareness within the scientific community and the manufacturing industry of the value of seeking alternatives to BPA for their indiscriminate use.

## Abbreviations

AML: acute myeloid leukemia
APLN: apelin
AR: androgen receptor
ATP: adenosine triphosphate
BC: breast cancer
BCL2: B cell lymphoma 2
BPA: bisphenol A
CC: cervical cancer
CDH2: cadherin 2
COX-2: cyclooxygenase 2
EC: endometrial cancer
EDCs: endocrine-disrupting chemicals
EGF: epidermal growth factor
EMT: epithelial-mesenchymal transition
ERK: extracellular signal-regulated kinases
ERα and β: estrogen receptors α and β
FN: fibronectin
GPER: G protein-coupled estrogen receptor
GPR: G protein-coupled receptor
GSK3 β: glycogen synthase kinase 3β
HCC: hepatocellular carcinoma
HOXB9: homeobox B9
IGF: insulin-like growth factor
IL6: interleukin 6
JAK: Janus kinase
LAMP3: lysosomal-associated membrane protein 3
LC: lung cancer
MAPK: mitogen-activated protein kinase
mERs: membrane estrogen receptors
miRNA: microRNA
MMP: mitochondrial membrane potential
NF-κB: nuclear factor kappa B
OC: oral cancer
OPC: oropharyngeal cancer
OVC: ovarian cancer
PARP: poly ADP ribose polymerase
PC: prostate cancer
PI3K: phosphoinositide-3-kinase
PPARγ: proliferator-activated receptor gamma
ROS: reactive oxygen species
SOD: superoxide dismutase
STAT3: signal transducer and activator of transcription 3
TAM: tamoxifen
TC: testicular cancer
TCs: thyroid cancer
TNF-α: tumor necrosis factor-α
UGTs: UDP-glucuronosyltransferases
VIM: vimentin

## References

[CR1] Abedelhaffez AS, El-Aziz EAA, Aziz MAA, Ahmed AM (2017). Lung injury induced by bisphenol A: a food contaminant, is ameliorated by selenium supplementation. Pathophysiology.

[CR2] Aboul Ezz HS, Khadrawy YA, Mourad IM (2015). The effect of bisphenol A on some oxidative stress parameters and acetylcholinesterase activity in the heart of male albino rats. Cytotechnology.

[CR3] Acconcia F, Pallottini V, Marino M (2015). Molecular mechanisms of action of BPA. Dose-Response.

[CR4] Adiga D, Eswaran S, Sriharikrishnaa S (2020). Role of epigenetic changes in reproductive inflammation and male infertility. Chem Biol Lett.

[CR5] Ajani MA, Salami A, Awolude OA, Oluwasola AO (2017). Hormone-receptor expression status of epithelial ovarian cancer in Ibadan, South-western Nigeria. Pan Afr Med J.

[CR6] Aquino CI, Troisi J, D’Antonio A (2019). Endometrial carcinoma and bisphenol A: a pilot case-control study. Biomed J Sci Tech Res.

[CR7] Bach DH, Park HJ, Lee SK (2018). The dual role of bone morphogenetic proteins in cancer. Mol Ther - Oncolytics.

[CR8] Balogh J, Victor D, Asham EH (2016). Hepatocellular carcinoma: a review. J Hepatocell Carcinoma.

[CR9] Barret J (2009). Trumped Treatment?: BPA blocks effects of breast cancer chemotherapy drugs. Environ Health Perspect.

[CR10] Bernstein L (2002). Epidemiology of endocrine-related risk factors for breast cancer. J Mammary Gland Biol Neoplasia.

[CR11] Bilancio A, Bontempo P, Di Donato M (2017). Bisphenol A induces cell cycle arrest in primary and prostate cancer cells through EGFR/ERK/p53 signaling pathway activation. Oncotarget.

[CR12] Bontempo P, Mita L, Doto A, Miceli M, Nebbioso A, Lepore I, Franci G, Menafra R, Carafa V, Conte M, de Bellis F, Manzo F, di Cerbo V, Benedetti R, D'Amato L, Marino M, Bolli A, del Pozzo G, Diano N, Portaccio M, Mita GD, Vietri MT, Cioffi M, Nola E, Dell'Aversana C, Sica V, Molinari A, Altucci L (2009). Molecular analysis of the apoptotic effects of BPA in acute myeloid leucemia cells. J Transl Med.

[CR13] Brett MR, Jennifer BP, Thomas AS (2017). Epidemiology of ovarian cancer: a review. Cancer Biol Med.

[CR14] Brooks RA, Fleming GF, Lastra RR, Lee NK, Moroney JW, Son CH, Tatebe K, Veneris JL (2019) Current recommendations and recent progress in endometrial cancer. CA Cancer J Clin:258–279. 10.3322/caac.21561 10.3322/caac.21561 31074865

[CR15] Camacho L, Lewis SM, Vanlandingham MM, Olson GR, Davis KJ, Patton RE, Twaddle NC, Doerge DR, Churchwell MI, Bryant MS, McLellen FM, Woodling KA, Felton RP, Maisha MP, Juliar BE, Gamboa da Costa G, Delclos KB (2019). A two-year toxicology study of bisphenol A (BPA) in Sprague-Dawley rats: CLARITY-BPA core study results. Food Chem Toxicol.

[CR16] Carbone C, Piro G, Simionato F, Ligorio F, Cremolini C, Loupakis F, Alì G, Rossini D, Merz V, Santoro R, Zecchetto C, Zanotto M, di Nicolantonio F, Bardelli A, Fontanini G, Tortora G, Melisi D (2017). Homeobox B9 mediates resistance to anti-VEGF Therapy in colorectal cancer patients. Clin Cancer Res.

[CR17] Cariati F, D’Uonno N, Borrillo F, Iervolino S, Galdiero G, Tomaiuolo R (2019). Bisphenol a: an emerging threat to male fertility. Reprod Biol Endocrinol.

[CR18] Chen ZJ, Yang XL, Liu H, Wei W, Zhang KS, Huang HB, Giesy JP, Liu HL, du J, Wang HS (2015). Bisphenol A modulates colorectal cancer protein profile and promotes the metastasis via induction of epithelial to mesenchymal transitions. Arch Toxicol.

[CR19] Cheong A, Johnson SA, Howald EC, Ellersieck MR, Camacho L, Lewis SM, Vanlandingham MM, Ying J, Ho SM, Rosenfeld CS (2018). Gene expression and DNA methylation changes in the hypothalamus and hippocampus of adult rats developmentally exposed to bisphenol A or ethinyl estradiol: a CLARITY-BPA consortium study. Epigenetics.

[CR20] Chevalier N, Bouskine A, Fenichel P (2012). Bisphenol A promotes testicular seminoma cell proliferation through GPER/GPR30. Int J Cancer.

[CR21] Chou W-C, Lee P-H, Tan Y-Y, Lin HC, Yang CW, Chen KH, Chuang CY (2017). An integrative transcriptomic analysis reveals bisphenol A exposure-induced dysregulation of microRNA expression in human endometrial cells. Toxicol Vitr.

[CR22] Cimmino I, Fiory F, Perruolo G, Miele C, Beguinot F, Formisano P, Oriente F (2020). Potential mechanisms of bisphenol A (BPA) contributing to human disease. Int J Mol Sci.

[CR23] Clément F, Xu X, Donini CF, Clément A, Omarjee S, Delay E, Treilleux I, Fervers B, le Romancer M, Cohen PA, Maguer-Satta V (2017). Long-term exposure to bisphenol A or benzo(a)pyrene alters the fate of human mammary epithelial stem cells in response to BMP2 and BMP4, by pre-activating BMP signaling. Cell Death Differ.

[CR24] da Silva MM, Xavier LLF, Gonçalves CFL, Santos-Silva AP, Paiva-Melo FD, de Freitas ML, Fortunato RS, Miranda-Alves L, Ferreira ACF (2018). Bisphenol A increases hydrogen peroxide generation by thyrocytes both in vivo and in vitro. Endocr Connect.

[CR25] Dairkee SH, Luciani-Torres MG, Moore DH, Goodson WH (2013). Bisphenol-A-induced inactivation of the p53 axis underlying deregulation of proliferation kinetics, and cell death in non-malignant human breast epithelial cells. Carcinogenesis.

[CR26] Davis AP, Grondin CJ, Johnson RJ, Sciaky D, Wiegers J, Wiegers TC, Mattingly CJ (2021). Comparative Toxicogenomics Database (CTD): update 2021. Nucleic Acids Res.

[CR27] Deb P, Bhan A, Hussain I, Ansari KI, Bobzean SA, Pandita TK, Perrotti LI, Mandal SS (2016). Endocrine disrupting chemical, bisphenol-A, induces breast cancer associated gene HOXB9 expression in vitro and in vivo. Gene.

[CR28] Delbès G, Levacher C, Habert R (2006). Estrogen effects on fetal and neonatal testicular development. Reproduction.

[CR29] den Boon JA, Pyeon D, Wang SS, Horswill M, Schiffman M, Sherman M, Zuna RE, Wang Z, Hewitt SM, Pearson R, Schott M, Chung L, He Q, Lambert P, Walker J, Newton MA, Wentzensen N, Ahlquist P (2015). Molecular transitions from papillomavirus infection to cervical precancer and cancer: role of stromal estrogen receptor signaling. Proc Natl Acad Sci U S A.

[CR30] den Braver-Sewradj SP, van Spronsen R, Hessel EVS (2020). Substitution of bisphenol A: a review of the carcinogenicity, reproductive toxicity, and endocrine disruption potential of alternative substances. Crit Rev Toxicol.

[CR31] Derouiche S, Warnier M, Mariot P, Gosset P, Mauroy B, Bonnal JL, Slomianny C, Delcourt P, Prevarskaya N, Roudbaraki M (2013). Bisphenol A stimulates human prostate cancer cell migration via remodelling of calcium signalling. Springerplus.

[CR32] Di Donato M, Cernera G, Giovannelli P (2017). Recent advances on bisphenol-A and endocrine disruptor effects on human prostate cancer. Mol Cell Endocrinol.

[CR33] Esplugas R, LLovet MI, Bellés M, Serra N, Vallvé JC, Domingo JL, Linares V (2018). Renal and hepatic effects following neonatal exposure to low doses of bisphenol-A and 137Cs. Food Chem Toxicol.

[CR34] Fatma Karaman E, Caglayan M, Sancar-Bas S, Ozal-Coskun C, Arda-Pirincci P, Ozden S (2019). Global and region-specific post-transcriptional and post-translational modifications of bisphenol A in human prostate cancer cells. Environ Pollut.

[CR35] Fenton SE (2006). Endocrine-disrupting compounds and mammary gland development: early exposure and later life consequences. Endocrinology.

[CR36] Fernandez S (2012). Expression and DNA methylation changes in human breast epithelial cells after bisphenol A exposure. Int J Oncol.

[CR37] Fic A, Jurković Mlakar S, Juvan P, Mlakar V, Marc J, Sollner Dolenc M, Broberg K, Peterlin Mašič L (2015). Genome-wide gene expression profiling of low-dose, long-term exposure of human osteosarcoma cells to bisphenol A and its analogs bisphenols AF and S. Toxicol Vitr.

[CR38] Gao H, Yang B, Li N (2015). Bisphenol A and hormone-associated cancers : current progress and perspectives. Medicine (Baltimore).

[CR39] Ge L-C, Chen Z-J, Liu H, Zhang KS, Su Q, Ma XY, Huang HB, Zhao ZD, Wang YY, Giesy JP, du J, Wang HS (2014). Signaling related with biphasic effects of bisphenol A (BPA) on Sertoli cell proliferation: a comparative proteomic analysis. Biochim Biophys Acta - Gen Subj.

[CR40] Genuis SJ, Beesoon S, Birkholz D, Lobo RA (2012). Human excretion of bisphenol A: Blood, Urine, and Sweat (BUS) Study. J Environ Public Health.

[CR41] Gertz J, Reddy TE, Varley KE, Garabedian MJ, Myers RM (2012). Genistein and bisphenol A exposure cause estrogen receptor 1 to bind thousands of sites in a cell type-specific manner. Genome Res.

[CR42] Ghebeh H, Al-Khaldi S, Olabi S (2014). Fascin is involved in the chemotherapeutic resistance of breast cancer cells predominantly via the PI3K/Akt pathway. Br J Cancer.

[CR43] Gómora MJ, Morales-Vásquez F, Pedernera E, Perez-Montiel D, López-Basave H, Villa AR, Hernández-Martínez A, Mena E, Mendez C (2018). Sexual steroid hormone receptors profiles of ovarian carcinoma in Mexican women. Endocr Connect.

[CR44] Hafezi SA, Abdel-Rahman WM (2019). The endocrine disruptor bisphenol A (BPA) exerts a wide range of effects in carcinogenesis and response to therapy. Curr Mol Pharmacol.

[CR45] Hall JM, Korach KS (2013). Endocrine disrupting chemicals promote the growth of ovarian cancer cells via the ER-CXCL12-CXCR4 signaling axis. Mol Carcinog.

[CR46] Hanafi NI, Kadir SHSA, Musa M (2019). Low concentration of bisphenol A induces proliferation of gastric cancer cells, HGC-27. J Teknol.

[CR47] Hassan ZK, Elobeid MA, Virk P, Omer SA, ElAmin M, Daghestani MH, AlOlayan EM (2012). Bisphenol A induces hepatotoxicity through oxidative stress in rat model. Oxid Med Cell Longev.

[CR48] Heindel JJ, Belcher S, Flaws JA, Prins GS, Ho SM, Mao J, Patisaul HB, Ricke W, Rosenfeld CS, Soto AM, vom Saal FS, Zoeller RT (2020). Data integration, analysis, and interpretation of eight academic CLARITY-BPA studies. Reprod Toxicol.

[CR49] Ho S-M, Tang W-Y, Belmonte de Frausto J, Prins GS (2006). Developmental exposure to estradiol and bisphenol A increases susceptibility to prostate carcinogenesis and epigenetically regulates phosphodiesterase type 4 variant 4. Cancer Res.

[CR50] Ho SM, Cheong A, Lam HM, Hu WY, Shi GB, Zhu X, Chen J, Zhang X, Medvedovic M, Leung YK, Prins GS (2015). Exposure of human prostaspheres to bisphenol a epigenetically regulates SNORD family noncoding RNAs via histone modification. Endocrinology.

[CR51] Hoffmann M, Fiedor E, Ptak A (2017). Bisphenol A and its derivatives tetrabromobisphenol A and tetrachlorobisphenol A induce apelin expression and secretion in ovarian cancer cells through a peroxisome proliferator-activated receptor gamma-dependent mechanism. Toxicol Lett.

[CR52] Hoque E (2019). Evaluation of bisphenol a induced effects on blood bio-chemical constituents and histo-structure of liver in Swiss albino mice and its ‘one health’ perspectives. J Vet Med One Heal Res.

[CR53] Huang DY, Zheng CC, Pan Q, Wu SS, Su X, Li L, Wu JH, Sun ZY (2018). Oral exposure of low-dose bisphenol A promotes proliferation of dorsolateral prostate and induces epithelial-mesenchymal transition in aged rats. Sci Rep.

[CR54] Huc L, Lemarié A, Guéraud F, Héliès-Toussaint C (2012). Low concentrations of bisphenol A induce lipid accumulation mediated by the production of reactive oxygen species in the mitochondria of HepG2 cells. Toxicol Vitr.

[CR55] Hui L, Li H, Lu G, Chen Z, Sun W, Shi Y, Fu Z, Huang B, Zhu X, Lu W, Xia D, Wu Y (2018). Low dose of bisphenol A modulates ovarian cancer gene expression profile and promotes epithelial to mesenchymal transition via canonical wnt pathway. Toxicol Sci.

[CR56] Ilagan Y, Mamillapalli R, Goetz LG (2017). Bisphenol-A exposure in utero programs a sexually dimorphic estrogenic state of hepatic metabolic gene expression. Reprod Toxicol.

[CR57] Inadera H (2015). Neurological effects of bisphenol A and its analogues. Int J Med Sci.

[CR58] Jammal M, Lima C, Murta E, Nomelini R (2017). Is ovarian cancer prevention currently still a recommendation of our grandparents?. Rev Bras Ginecol e Obs / RBGO Gynecol Obstet.

[CR59] Januchowski R, Świerczewska M, Sterzyńska K, Wojtowicz K, Nowicki M, Zabel M (2016). Increased expression of several collagen genes is associated with drug resistance in ovarian cancer cell lines. J Cancer.

[CR60] Jeong JS, Nam KT, Lee B, Pamungkas AD, Song D, Kim M, Yu WJ, Lee J, Jee S, Park YH, Lim KM (2017). Low-dose bisphenol A increases bile duct proliferation in juvenile rats: a possible evidence for risk of liver cancer in the exposed population?. Biomol Ther.

[CR61] Jia J, Tian Q, Liu Y, Shao ZW, Yang SH (2013). Interactive effect of bisphenol A (BPA) exposure with -22G/C polymorphism in LOX gene on the risk of osteosarcoma. Asian Pacific J Cancer Prev.

[CR62] Jiang XW, Ge CX, Ge SF, Li WL (2016) Potential bisphenol AF-irinotecan interaction during the treatment of renal cell carcinoma. Lat Am J Pharm 35:2036–2040

[CR63] Kawai K, Nozaki T, Nishikata H, Aou S, Takii M, Kubo C (2003). Aggressive behavior and serum testosterone concentration during the maturation process of male mice: the effects of fetal exposure to bisphenol A. Environ Health Perspect.

[CR64] Kidani T, Yasuda R, Miyawaki J et al (2017) Bisphenol a inhibits cell proliferation and reduces the motile potential of murine LM8 osteosarcoma cells. Anticancer Res 37:1711–1722. 10.21873/anticanres.11503 10.21873/anticanres.11503 28373433

[CR65] Kim Y-S, Hwang K-A, Hyun S-H, Nam KH, Lee CK, Choi KC (2015). Bisphenol A and nonylphenol have the potential to stimulate the migration of ovarian cancer cells by inducing epithelial–mesenchymal transition via an estrogen receptor dependent pathway. Chem Res Toxicol.

[CR66] Kim J, Choi H, Lee H (2017). Effects of bisphenol compounds on the growth and epithelial mesenchymal transition of MCF-7 CV human breast cancer cells. J Biomed Res.

[CR67] Klotz DM, Hewitt SC, Korach KS, Diaugustine RP (2000). Activation of a uterine insulin-like growth factor I signaling pathway by clinical and environmental estrogens: requirement of estrogen receptor-α. Endocrinology.

[CR68] Konieczna A, Rachoń D, Owczarek K, Kubica P, Kowalewska A, Kudłak B, Wasik A, Namieśnik J (2018). Serum bisphenol A concentrations correlate with serum testosterone levels in women with polycystic ovary syndrome. Reprod Toxicol.

[CR69] Kubwabo C, Kosarac I, Stewart B, Gauthier BR, Lalonde K, Lalonde PJ (2009). Migration of bisphenol A from plastic baby bottles, baby bottle liners and reusable polycarbonate drinking bottles. Food Addit Contam - Part A Chem Anal Control Expo Risk Assess.

[CR70] Kurebayashi H (2003). Disposition of a low dose of 14C-bisphenol A in male rats and its main biliary excretion as BPA glucuronide. Toxicol Sci.

[CR71] Lan HC, Lin IW, Yang ZJ, Lin JH (2015) Low-dose bisphenol A activates Cyp11a1 gene expression and corticosterone secretion in adrenal gland via the JNK signaling pathway. Toxicol Sci 148:26–34. 10.1093/toxsci/kfv162 10.1093/toxsci/kfv162 26209791

[CR72] LaPensee EW, Tuttle TR, Fox SR, Ben-Jonathan N (2009). Bisphenol A at low nanomolar doses confers chemoresistance in estrogen receptor-α–positive and –negative breast cancer cells. Environ Health Perspect.

[CR73] Lee H-R, Kim T-H, Choi K-C (2012). Functions and physiological roles of two types of estrogen receptors, ERα and ERβ, identified by estrogen receptor knockout mouse. Lab Anim Res.

[CR74] Lee H-S, Kang Y, Tae K, Bae GU, Park JY, Cho YH, Yang M (2018). Proteomic biomarkers for bisphenol A–early exposure and women’s thyroid cancer. Cancer Res Treat.

[CR75] Li S, Wang B, Tang Q, Liu J, Yang X (2017). Bisphenol A triggers proliferation and migration of laryngeal squamous cell carcinoma via GPER mediated upregulation of IL-6. Cell Biochem Funct.

[CR76] Li J, Bach A, Crawford RB, Phadnis-Moghe AS, Chen W, D’Ingillo S, Kovalova N, Suarez-Martinez JE, Zhou J, Kaplan BLF, Kaminski NE (2018). CLARITY-BPA: effects of chronic Bisphenol A exposure on the immune system: Part 1 - Quantification of the relative number and proportion of leukocyte populations in the spleen and thymus. Toxicology.

[CR77] Li L, Ying Y, Zhang C, Wang W, Li Y, Feng Y, Liang J, Song H, Wang Y (2019). Bisphenol A exposure and risk of thyroid nodules in Chinese women: A case-control study. Environ Int.

[CR78] Li J, Ji Z, Luo X, Li Y, Yuan P, Long J, Shen N, Lu Q, Zeng Q, Zhong R, Shen Y, Cheng L (2020). Urinary bisphenol A and its interaction with ESR1 genetic polymorphism associated with non-small cell lung cancer: findings from a case-control study in Chinese population. Chemosphere.

[CR79] Lin H, Li H, Lu G (2018). Low dose of bisphenol a modulates ovarian cancer gene expression profile and promotes epithelial to mesenchymal transition via canonical wnt pathway. Toxicol Sci.

[CR80] Lind T, Lejonklou MH, Dunder L, Kushnir MM, Öhman-Mägi C, Larsson S, Melhus H, Lind PM (2019). Developmental low-dose exposure to bisphenol A induces chronic inflammation, bone marrow fibrosis and reduces bone stiffness in female rat offspring only. Environ Res.

[CR81] Ma XF, Zhang J, Shuai HL, Guan BZ, Luo X, Yan RL (2015). IKKβ/NF-κB mediated the low doses of bisphenol A induced migration of cervical cancer cells. Arch Biochem Biophys.

[CR82] Mandrup K, Boberg J, Isling LK, Christiansen S, Hass U (2016). Low-dose effects of bisphenol A on mammary gland development in rats. Andrology.

[CR83] Medwid S, Guan H, Yang K (2018) Bisphenol A stimulates adrenal cortical cell proliferation via ERβ-mediated activation of the sonic hedgehog signalling pathway. J Steroid Biochem Mol Biol 178:254–262. 10.1016/j.jsbmb.2018.01.004 10.1016/j.jsbmb.2018.01.004 29307715

[CR84] Moon MK, Kim MJ, Jung IK, Koo YD, Ann HY, Lee KJ, Kim SH, Yoon YC, Cho BJ, Park KS, Jang HC, Park YJ (2012). Bisphenol A impairs mitochondrial function in the liver at doses below the no observed adverse effect level. J Korean Med Sci.

[CR85] Naciff JM (2003). Gene Expression Profile Induced by 17alpha-Ethynyl Estradiol in the Prepubertal Female Reproductive System of the Rat. Toxicol Sci.

[CR86] Nava-Castro KE, Ramírez-Nieto R, Méndez-García LA, Girón-Pérez MI, Segovia-Mendoza M, Navidad-Murrieta MS, Morales Montor J (2019). Environmental pollution as a risk factor in testicular tumour development: focus on the interaction between bisphenol A and the associated immune response. Int J Environ Res Public Health.

[CR87] Newbold RR, Jefferson WN, Padilla-Banks E (2007). Long-term adverse effects of neonatal exposure to bisphenol A on the murine female reproductive tract. Reprod Toxicol.

[CR88] Newbold RR, Jefferson WN, Padilla-Banks E (2009). Prenatal exposure to bisphenol A at environmentally relevant doses adversely affects the murine female reproductive tract later in life. Environ Health Perspect.

[CR89] Nomiri S, Hoshyar R, Ambrosino C, Tyler CR, Mansouri B (2019). A mini review of bisphenol A (BPA) effects on cancer-related cellular signaling pathways. Environ Sci Pollut Res.

[CR90] Paris F, Balaguer P, Térouanne B, Servant N, Lacoste C, Cravedi JP, Nicolas JC, Sultan C (2002). Phenylphenols, biphenols, bisphenol-A and 4-tert-octylphenol exhibit α and β estrogen activities and antiandrogen activity in reporter cell lines. Mol Cell Endocrinol.

[CR91] Park M-A, Choi K-C (2014). Effects of 4-nonylphenol and bisphenol A on stimulation of cell growth via disruption of the transforming growth factor-β signaling pathway in ovarian cancer models. Chem Res Toxicol.

[CR92] Peretz J, Craig ZR, Flaws JA (2012). Bisphenol A inhibits follicle growth and induces atresia in cultured mouse antral follicles independently of the genomic estrogenic pathway1. Biol Reprod.

[CR93] Pfeifer D, Chung YM, Hu MC-T (2015). Effects of low-dose bisphenol A on DNA damage and proliferation of breast cells: the role of c-Myc. Environ Health Perspect.

[CR94] Pivonello C, Muscogiuri G, Nardone A, Garifalos F, Provvisiero DP, Verde N, de Angelis C, Conforti A, Piscopo M, Auriemma RS, Colao A, Pivonello R (2020). Bisphenol A: an emerging threat to female fertility. Reprod Biol Endocrinol.

[CR95] Prins GS, Hu W-Y, Shi G-B, Hu DP, Majumdar S, Li G, Huang K, Nelles JL, Ho SM, Walker CL, Kajdacsy-Balla A, van Breemen RB (2014). Bisphenol A promotes human prostate stem-progenitor cell self-renewal and increases in vivo carcinogenesis in human prostate epithelium. Endocrinology.

[CR96] Prins GS, Calderon-Gierszal EL, Hu W-Y (2015). Stem cells as hormone targets that lead to increased cancer susceptibility. Endocrinology.

[CR97] Prins GS, Ye S-H, Birch L, Zhang X, Cheong A, Lin H, Calderon-Gierszal E, Groen J, Hu WY, Ho SM, van Breemen RB (2017). Prostate cancer risk and DNA methylation Signatures in aging rats following developmental BPA exposure: a dose–response analysis. Environ Health Perspect.

[CR98] Prins GS, Hu W-Y, Xie L, Shi GB, Hu DP, Birch L, Bosland MC (2018). Evaluation of bisphenol A (BPA) exposures on prostate stem cell homeostasis and prostate cancer risk in the NCTR-Sprague-Dawley rat: an NIEHS/FDA CLARITY-BPA consortium study. Environ Health Perspect.

[CR99] Prossnitz ER, Barton M (2011). The G protein-coupled estrogen receptor GPER in health and disease. Nat Rev Endocrinol.

[CR100] Ptak A, Gregoraszczuk EL (2012). Bisphenol A induces leptin receptor expression, creating more binding sites for leptin, and activates the JAK/Stat, MAPK/ERK and PI3K/Akt signalling pathways in human ovarian cancer cell. Toxicol Lett.

[CR101] Ptak A, Wróbel A, Gregoraszczuk EL (2011). Effect of bisphenol-A on the expression of selected genes involved in cell cycle and apoptosis in the OVCAR-3 cell line. Toxicol Lett.

[CR102] Ptak A, Hoffmann M, Gruca I, Barć J (2014). Bisphenol A induce ovarian cancer cell migration via the MAPK and PI3K/Akt signalling pathways. Toxicol Lett.

[CR103] Pupo M, Pisano A, Lappano R, Santolla MF, de Francesco EM, Abonante S, Rosano C, Maggiolini M (2012). Bisphenol A Induces gene expression changes and proliferative effects through GPER in breast cancer cells and cancer-associated fibroblasts. Environ Health Perspect.

[CR104] Qu W, Zhao Z, Chen S, Zhang L, Wu D, Chen Z (2018). Bisphenol A suppresses proliferation and induces apoptosis in colonic epithelial cells through mitochondrial and MAPK/AKT pathways. Life Sci.

[CR105] Rachoń D (2015). Endocrine disrupting chemicals (EDCs) and female cancer: informing the patients. Rev Endocr Metab Disord.

[CR106] Rachoń D, Menche A, Vortherms T, Seidlová-Wuttke D, Wuttke W (2008). Effects of dietary equol administration on the mammary gland in ovariectomized Sprague-Dawley rats. Menopause.

[CR107] Reed CE, Fenton SE (2013). Exposure to diethylstilbestrol during sensitive life stages: a legacy of heritable health effects. Birth Defects Res Part C - Embryo Today Rev.

[CR108] Renaud L, da Silveira WA, Hazard ES (2017). The plasticizer bisphenol A perturbs the hepatic epigenome: a systems level analysis of the miRNome. Genes (Basel).

[CR109] Ryszawy D, Pudełek M, Kochanowski P, Janik-Olchawa N, Bogusz J, Rąpała M, Koczurkiewicz P, Mikołajczyk J, Borek I, Kędracka-Krok S, Karnas E, Zuba-Surma E, Madeja Z, Czyż J (2019). High bisphenol A concentrations augment the invasiveness of tumor cells through Snail-1/Cx43/ERRγ-dependent epithelial-mesenchymal transition. Toxicol Vitr.

[CR110] Sangai NP, Verma RJ, Trivedi MH (2014). Testing the efficacy of quercetin in mitigating bisphenol A toxicity in liver and kidney of mice. Toxicol Ind Health.

[CR111] Sauer SJ, Tarpley M, Shah I, Save AV, Lyerly HK, Patierno SR, Williams KP, Devi GR (2017). Bisphenol A activates EGFR and ERK promoting proliferation, tumor spheroid formation and resistance to EGFR pathway inhibition in estrogen receptor-negative inflammatory breast cancer cells. Carcinogenesis.

[CR112] Seachrist DD, Bonk KW, Ho S-M, Prins GS, Soto AM, Keri RA (2016). A review of the carcinogenic potential of bisphenol A. Reprod Toxicol.

[CR113] Şenyildiz M, Özden S (2015). Alteration in global DNA methylation after bisphenol a exposure in MCF-7 cells. J Pharm Istanbul Univ.

[CR114] Şenyıldız M, Karaman EF, Baş SS (2016). Alteration on global and gene-spesific DNA methylation and global histone modifications in HepG2 cells in response to BPA. J Fac Pharm Istanbul / İstanbul Ecz Fak Derg.

[CR115] Senyildiz M, Karaman EF, Bas SS, Pirincci PA, Ozden S (2017). Effects of BPA on global DNA methylation and global histone 3 lysine modifications in SH-SY5Y cells: an epigenetic mechanism linking the regulation of chromatin modifiying genes. Toxicol Vitr.

[CR116] Sheng ZG, Huang W, Liu YX, Zhu BZ (2013). Bisphenol A at a low concentration boosts mouse spermatogonial cell proliferation by inducing the G protein-coupled receptor 30 expression. Toxicol Appl Pharmacol.

[CR117] Shi XY, Wang Z, Liu L, Feng LM, Li N, Liu S, Gao H (2017). Low concentrations of bisphenol A promote human ovarian cancer cell proliferation and glycolysis-based metabolism through the estrogen receptor-Α pathway. Chemosphere.

[CR118] Song H, Zhang T, Yang P, Li M, Yang Y, Wang Y, du J, Pan K, Zhang K (2015). Low doses of bisphenol A stimulate the proliferation of breast cancer cells via ERK1/2/ERRγ signals. Toxicol Vitr.

[CR119] Tarapore P, Ying J, Ouyang B, Burke B, Bracken B, Ho SM (2014). Exposure to bisphenol A correlates with early-onset prostate cancer and promotes centrosome amplification and anchorage-independent growth in vitro. PLoS One.

[CR120] Terasaka H, Kadoma Y, Sakagami H, Fujisawa S (2005). Cytotoxicity and apoptosis-inducing activity of bisphenol A and hydroquinone in HL-60 cells. Anticancer Res.

[CR121] Thoene M, Rytel L, Dzika E, Włodarczyk A, Kruminis-Kaszkiel E, Konrad P, Wojtkiewicz J (2017). Bisphenol A causes liver damage and selectively alters the neurochemical coding of intrahepatic parasympathetic nerves in juvenile porcine models under physiological conditions. Int J Mol Sci.

[CR122] Thongkorn S, Kanlayaprasit S, Jindatip D, Tencomnao T, Hu VW, Sarachana T (2019). Sex differences in the effects of prenatal bisphenol A exposure on genes associated with autism spectrum disorder in the hippocampus. Sci Rep.

[CR123] Tournaire M, Devouche E, Espié M, Asselain B, Levadou A, Cabau A, Dunbavand A, Grosclaude P, Epelboin S (2015). Cancer risk in women exposed to diethylstilbestrol in utero. Therapie.

[CR124] Tsai W-T (2006). Human health risk on environmental exposure to bisphenol-A: a review. J Environ Sci Heal Part C.

[CR125] Vandenberg LN, Chahoud I, Heindel JJ, Padmanabhan V, Paumgartten FJR, Schoenfelder G (2010). Urinary, circulating, and tissue biomonitoring studies indicate widespread exposure to bisphenol A. Environ Health Perspect.

[CR126] Wang D, Hu L, Zhang G, Zhang L, Chen C (2010). G protein-coupled receptor 30 in tumor development. Endocrine.

[CR127] Wang K-H, Kao A-P, Chang C-C, Lin TC, Kuo TC (2013). Bisphenol A at environmentally relevant doses induces cyclooxygenase-2 expression and promotes invasion of human mesenchymal stem cells derived from uterine myoma tissue. Taiwan J Obstet Gynecol.

[CR128] Wang KH, Kao AP, Chang CC, Lin TC, Kuo TC (2015). Bisphenol A-induced epithelial to mesenchymal transition is mediated by cyclooxygenase-2 up-regulation in human endometrial carcinoma cells. Reprod Toxicol.

[CR129] Wang Z, Liu H, Liu S (2017). Low-dose bisphenol A exposure: a seemingly instigating carcinogenic effect on breast cancer. Adv Sci.

[CR130] Wang K, Zhao Z, Ji W (2019). Bisphenol A induces apoptosis, oxidative stress and inflammatory response in colon and liver of mice in a mitochondria-dependent manner. Biomed Pharmacother.

[CR131] Weinhouse C, Anderson OS, Bergin IL, Vandenbergh DJ, Gyekis JP, Dingman MA, Yang J, Dolinoy DC (2014). Dose-dependent incidence of hepatic tumors in adult mice following perinatal exposure to bisphenol A. Environ Health Perspect.

[CR132] Weng YI, Hsu PY, Liyanarachchi S, Liu J, Deatherage DE, Huang YW, Zuo T, Rodriguez B, Lin CH, Cheng AL, Huang TH (2010). Epigenetic influences of low-dose bisphenol A in primary human breast epithelial cells. Toxicol Appl Pharmacol.

[CR133] Wetherill YB, Hess-Wilson JK, Comstock CES, Shah SA, Buncher CR, Sallans L, Limbach PA, Schwemberger S, Babcock GF, Knudsen KE (2006). Bisphenol A facilitates bypass of androgen ablation therapy in prostate cancer. Mol Cancer Ther.

[CR134] Xi W, Wan HT, Zhao YG, Wong MH, Giesy JP, Wong CKC (2012). Effects of perinatal exposure to bisphenol A and di(2-ethylhexyl)-phthalate on gonadal development of male mice. Environ Sci Pollut Res.

[CR135] Xiong Y, Wen X, Liu H, Zhang M, Zhang Y (2020). Bisphenol a affects endometrial stromal cells decidualization, involvement of epigenetic regulation. J Steroid Biochem Mol Biol.

[CR136] Yaguchi T (2019). The endocrine disruptor bisphenol A promotes nuclear ERRγ translocation, facilitating cell proliferation of Grade I endometrial cancer cells via EGF-dependent and EGF-independent pathways. Mol Cell Biochem.

[CR137] Zhang KS, Chen HQ, Chen YS, Qiu KF, Zheng XB, Li GC, Yang HD, Wen CJ (2014). Bisphenol A stimulates human lung cancer cell migration via upregulation of matrix metalloproteinases by GPER/EGFR/ERK1/2 signal pathway. Biomed Pharmacother.

[CR138] Zhang B, Chen L, Bao Q, Zheng X (2016). Upregulation of fibronectin, vitronectin and claudin-7 in cervical cancer. Int J Clin Exp Med.

[CR139] Zhang J, Zhou C, Jiang H, Liang L, Shi W, Zhang Q, Sun P, Xiang R, Wang Y, Yang S (2017). ZEB1 induces ER-α promoter hypermethylation and confers antiestrogen resistance in breast cancer. Cell Death Dis.

[CR140] Zhang S, Li J, Fan J, Wu X (2020). Bisphenol A triggers the malignancy of acute myeloid leukemia cells via regulation of IL-4 and IL-6. J Biochem Mol Toxicol.

[CR141] Zhou Z, Zhang J, Jiang F, Xie Y, Zhang X, Jiang L (2017). Higher urinary bisphenol A concentration and excessive iodine intake are associated with nodular goiter and papillary thyroid carcinoma. Biosci Rep.

